# Restorative Effects of Synbiotics on Colonic Ultrastructure and Oxidative Stress in Dogs with Chronic Enteropathy

**DOI:** 10.3390/antiox14060727

**Published:** 2025-06-13

**Authors:** Dipak Kumar Sahoo, Tracey Stewart, Emily M. Lindgreen, Bhakti Patel, Ashish Patel, Jigneshkumar N. Trivedi, Valerie Parker, Adam J. Rudinsky, Jenessa A. Winston, Agnes Bourgois-Mochel, Jonathan P. Mochel, Karin Allenspach, Romy M. Heilmann, Albert E. Jergens

**Affiliations:** 1 Department of Veterinary Clinical Sciences, College of Veterinary Medicine, Iowa State University, Ames, IA 50011, USAkarin.allenspach@uga.edu (K.A.); 2 Office of Biotechnology, Roy J Carver High Resolution Microscopy Facility, Iowa State University, Ames, IA 50011, USA; 3 Department of Life Sciences, Hemchandracharya North Gujarat University, Patan 384265, Gujarat, Indiauni.ashish@gmail.com (A.P.); jntrivedi26@yahoo.co.in (J.N.T.); 4 Department of Veterinary Clinical Sciences, College of Veterinary Medicine, The Ohio State University, Columbus, OH 43210, USA; 5 Department of Pathology, Precision One Health Initiative, University of Georgia, Athens, GA 30602, USA; jpmochel@uga.edu; 6 Department for Small Animals, College of Veterinary Medicine, Leipzig University, DE-04103 Leipzig, Germany; romy.heilmann@kleintierklinik.uni-leipzig.de

**Keywords:** canine chronic enteropathy, cell signaling, electron microscopy, endoplasmic reticulum, gene expression, mitochondria, oxidative stress, synbiotic treatment, ultrastructure

## Abstract

Synbiotics can be used to reduce intestinal inflammation and mitigate dysbiosis in dogs with chronic inflammatory enteropathy (CIE). Prior research has not assessed the colonic mucosal ultrastructure of dogs with active CIE treated with synbiotics, nor has it determined a possible association between morphologic injury and signaling pathways. Twenty client-owned dogs diagnosed with CIE were randomized to receive either a hydrolyzed diet (placebo; PL) or a hydrolyzed diet supplemented with synbiotic-IgY (SYN) for 6 weeks. Endoscopic biopsies of the colon were obtained for histopathologic, ultrastructural, and molecular analyses and were compared before and after treatment. Using transmission electron microscopy (TEM), an analysis of the ultrastructural alterations in microvilli length (MVL), mitochondria (MITO), and rough endoplasmic reticulum (ER) was compared between treatment groups. To explore potential signaling pathways that might modulate MITO and ER stress, a transcriptomic analysis was also performed. The degree of mucosal ultrastructural pathology differed among individual dogs before and after treatment. Morphologic alterations in enterocytes, MVL, MITO, and ER were detected without significant differences between PL and SYN dogs prior to treatment. Notable changes in ultrastructural alterations were identified post-treatment, with SYN-treated dogs exhibiting significant improvement in MVL, MITO, and ER injury scores compared to PL-treated dogs. Transcriptomic profiling showed many pathways and key genes to be associated with MITO and ER injury. Multiple signaling pathways and their associated genes with protective effects, including fibroblast growth factor 2 (*FGF2*), fibroblast growth factor 7 (*FGF7*), fibroblast growth factor 10 (*FGF10*), synaptic Ras GTPase activating protein 1 (*SynGAP1*), RAS guanyl releasing protein 2 (*RASGRP2*), RAS guanyl releasing protein 3 (*RASGRP3*), thrombospondin 1 (*THBS1*), colony stimulating factor 1 (*CSF1*), colony stimulating factor 3 (*CSF3*), interleukin 21 receptor (*IL21R*), collagen type VI alpha 6 chain (*COL6A6*), ectodysplasin A receptor (*EDAR*), forkhead box P3 (*FoxP3*), follistatin (*FST*), gremlin 1 (*GREM1*), myocyte enhancer factor 2B (*MEF2B*), neuregulin 1 (*NRG1*), collagen type I alpha 1 chain (*COL1A1*), hepatocyte growth factor (*HGF*), 5-hydroxytryptamine receptor 7 (*HTR7*), and platelet derived growth factor receptor beta (*PDGFR-β*), were upregulated with SYN treatment. Differential gene expression was associated with improved MITO and ER ultrastructural integrity and a reduction in oxidative stress. Conversely, other genes, such as protein kinase cAMP-activated catalytic subunit beta (*PRKACB*), phospholipase A2 group XIIB (*PLA2G12B*), calmodulin 1 (*CALM1*), calmodulin 2 (*CALM2*), and interleukin-18 (*IL18*), which have harmful effects, were downregulated following SYN treatment. In dogs treated with PL, genes including *PRKACB* and *CALM2* were upregulated, while other genes, such as *FGF2*, *FGF10*, *SynGAP1*, *RASGRP2*, *RASGRP3*, and *IL21R*, were downregulated. Dogs with CIE have colonic ultrastructural pathology at diagnosis, which improves following synbiotic treatment. Ultrastructural improvement is associated with an upregulation of protective genes and a downregulation of harmful genes that mediate their effects through multiple signaling pathways.

## 1. Introduction

Canine chronic inflammatory enteropathy (CIE) refers to a group of gastrointestinal (GI) disorders characterized by persistent or intermittent GI signs and mucosal inflammation [1,2]. Clinical signs of diarrhea, vomiting, alterations in appetite, and/or weight loss are observed depending on which segment of the GI tract is involved and the extent of mucosal inflammation. While the cause of CIE is unknown, it is believed to be a multifactorial disorder involving host immunity, genetic variability, and environmental factors (diet and microbiome) [1]. The interplay between these factors results in heterogeneous disease expression, where clinical phenotypes, including food-responsive enteropathy (FRE), steroid- or immunosuppressant-responsive enteropathy (SRE/IRE), and non-responsive enteropathy (NRE), are recognized following treatment trials [1,2,3]. As intestinal dysbiosis has been extensively reported in dogs with CIE [4], probiotics, prebiotics, or synbiotics may be used to reduce intestinal inflammation and promote the recovery of microbial imbalances [5,6,7,8,9].

Endoscopy with histopathologic evaluation may be used to confirm the presence and extent of mucosal inflammation in dogs with CIE. However, even when using standardized grading criteria for defining intestinal inflammation [10], there can be discordance between clinical disease severity and histopathologic inflammation [11]. A simplified pathologic model using the World Small Animal Veterinary Association (WSAVA) criteria has shown the improved consistency of interpretations among pathologists [12]. Compared with light microscopy, electron microscopy (EM) enables the evaluation of intestinal mucosal surfaces using a much higher magnification to assess the internal and external ultrastructure of epithelial cells [13,14,15]. In one study, scanning electron microscopy (SEM) revealed ultrastructural lesions in 70% of children with celiac disease having a normal histology following long-term dietary therapy [16]. Different ultrastructural lesions involving the luminal epithelium have also been observed in humans with inflammatory bowel disease (IBD), including both Crohn’s disease (CD) [15,17,18,19,20,21,22,23,24] and ulcerative colitis (UC) [15,20,21,25,26,27]. There is a single report describing the pre- and post-treatment ultrastructural changes to duodenal enterocytes in dogs with FRE [28].

Several studies demonstrate the beneficial effects of prebiotics, probiotics, and postbiotics in human IBD [29]. Synbiotics, comprising probiotic and prebiotic components, can provide antioxidant activities and other health benefits to the host [29,30,31,32,33]. Synbiotics have been shown to preserve healthy GI microbiota, strengthen the intestinal mucosal barrier, improve immune tolerance, and modulate the pro-inflammatory response [29,30]. In addition, synbiotics exhibit several antioxidant properties by activating and translocating nuclear factor erythroid 2-related factor 2 (Nrf2). Moreover, they can stimulate the expression of the antioxidant defense enzymatic system, including superoxide dismutase (SOD), catalase, and glutathione peroxidase (GPx) activities; generate essential antioxidant molecules like glutathione (GSH); and neutralize the formation of ROS [29,30,32,34,35,36]. In the current study, we aimed to characterize the ultrastructural alterations in the colonic mucosa of dogs with CIE at diagnosis and following synbiotic treatment utilizing transmission electron microscopy (TEM). Furthermore, we compared ultrastructural changes in colonocytes to functional changes in genes involved with oxidative stress (OS) in inflamed mucosa using transcriptomics and pathway enrichment analysis, as well as OS parameters.

## 2. Materials and Methods

### 2.1. Ethical Concerns

The animal use/clinical trial protocol was reviewed and approved by the Institutional Animal Care and Use Committee (IACUC) of Iowa State University (ISU) (IACUC #19-158) and Ohio State University (IACUC-2019A00000100), and all pet owners gave written informed consent prior to their dog’s enrollment.

### 2.2. Study Design

The clinical trial was a prospective, randomized, double-blind, and placebo-controlled study with a six-week duration, which was performed at Iowa State University from December 2018 to December 2021 [9]. Sample size calculations were performed using previously published data showing that treatment with diet alone results in remission rates of approximately 50–60% in dogs with CIE [37,38]. A minimum significant difference in outcome between the two treatment groups was estimated at 25%. Therefore, a remission rate of 75% or greater was anticipated in supplement-treated dogs. The randomization of 15 dogs per group was calculated to provide a power of 80% to detect treatment differences at the 0.05 significance level. However, due to limited enrollment during the COVID-19 pandemic, only 20 dogs completed the trial [9]. In the clinical trial, these twenty dogs were randomly assigned to one of two treatment groups: a synbiotic (SYN; Intesto-Guard™, IG Biosciences, Newton, IA, USA) group or a control (placebo, PL) group. All dogs were fed a hydrolyzed protein diet for the completion of the treatment schedule (42 days). Colon biopsies were obtained by endoscopy at diagnosis (day 0) and following six weeks of treatment (day 42). Following endoscopic collection, mucosal biopsies were immediately immersed in a 1% paraformaldehyde and 3% glutaraldehyde fixative solution in preparation for EM. Another set of colon biopsies was promptly placed into vials with RNA*later* Stabilization Solution (ThermoFisher Scientific, San Jose, CA, USA) and preserved at −80 °C until RNA extraction. Archived tissues from five dogs in each treatment group were investigated for changes in colonic ultrastructure and transcriptomic profiles pre- versus post-treatment. For transcriptomics analysis, the minimum number of samples utilized for the current investigation was determined by a prior study, which suggested that at a sequencing depth of approximately 20 million reads for protein-coding mRNA, at least five samples need to be used to reduce statistical power disparity [39].

### 2.3. Transmission Electron Microscopy (TEM)

Canine colon biopsies were taken from the 1% paraformaldehyde/3% glutaraldehyde solution in a 0.1M sodium cacodylate buffer (pH 7.2) for standard processing procedures. Samples underwent a washing process in a 0.1M sodium cacodylate buffer and were subsequently post-fixed with 1% osmium tetroxide in the same buffer for a duration of 1 h at room temperature. Samples were then washed with deionized water and were subsequently stained en bloc with 2% uranyl acetate solution for a duration of 1 h. This was followed by a washing process in deionized water and dehydration using a graded series of ethanol concentrations (25, 50, 70, 85, 95, and 100% *v*/*v*). Samples underwent additional dehydration using three changes of pure acetone and were subsequently infiltrated with EmBed 812 formula (hard) for EPON epoxy resin (Electron Microscopy Sciences, Hatfield, PA, USA). This process involved the graded ratios of resin to acetone until complete infiltration with pure epoxy resin was achieved (3:1, 1:1, 1:3, pure) for 6–12 h for each step. Biopsies were positioned in Beem capsule lids to ensure proper orientation for cross-sectioning and underwent polymerization at 70 °C for a duration of 48 h. Thick sections (1.5 μm) were prepared utilizing a Leica UC6 ultramicrotome (Leica Microsystems, Buffalo Grove, IL, USA) and subsequently stained with methylene blue and basic fuchsin. Thin sections were prepared at a thickness of 50 nm and subsequently placed onto single-slot carbon film grids utilizing a Leica UC6 ultramicrotome (Leica Microsystems Inc., Deerfield, IL, USA). TEM images were obtained utilizing a 200 kV JEOL JSM 2100 scanning transmission electron microscope (Japan Electron Optics Laboratories, Peabody, MA, USA) equipped with a GATAN One View 4K camera (Gatan Inc., Pleasanton, CA, USA).

### 2.4. Morphologic Criteria for Assessing Ultrastructural Changes

**Figure 1:**
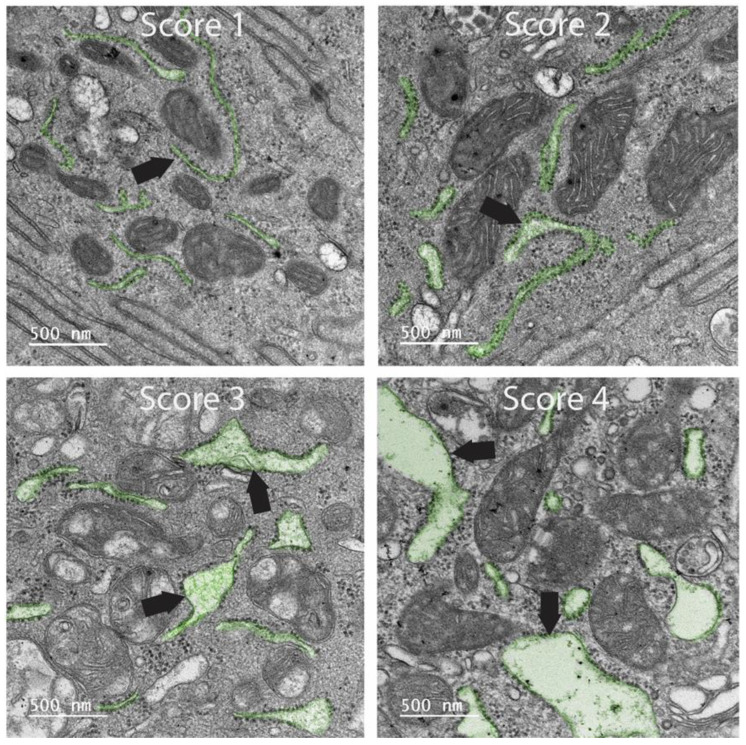
Transmission electron microscopy (TEM) showing the ER scoring of colonocytes. Green highlights denote ER. ER score 1: normal appearance (arrowhead); ER score 2: some normal, some distended (arrowhead); ER score 3: few normal and many larger distended (arrowheads); ER score 4: the majority are distended and blown out (arrowheads).

**Figure 2:**
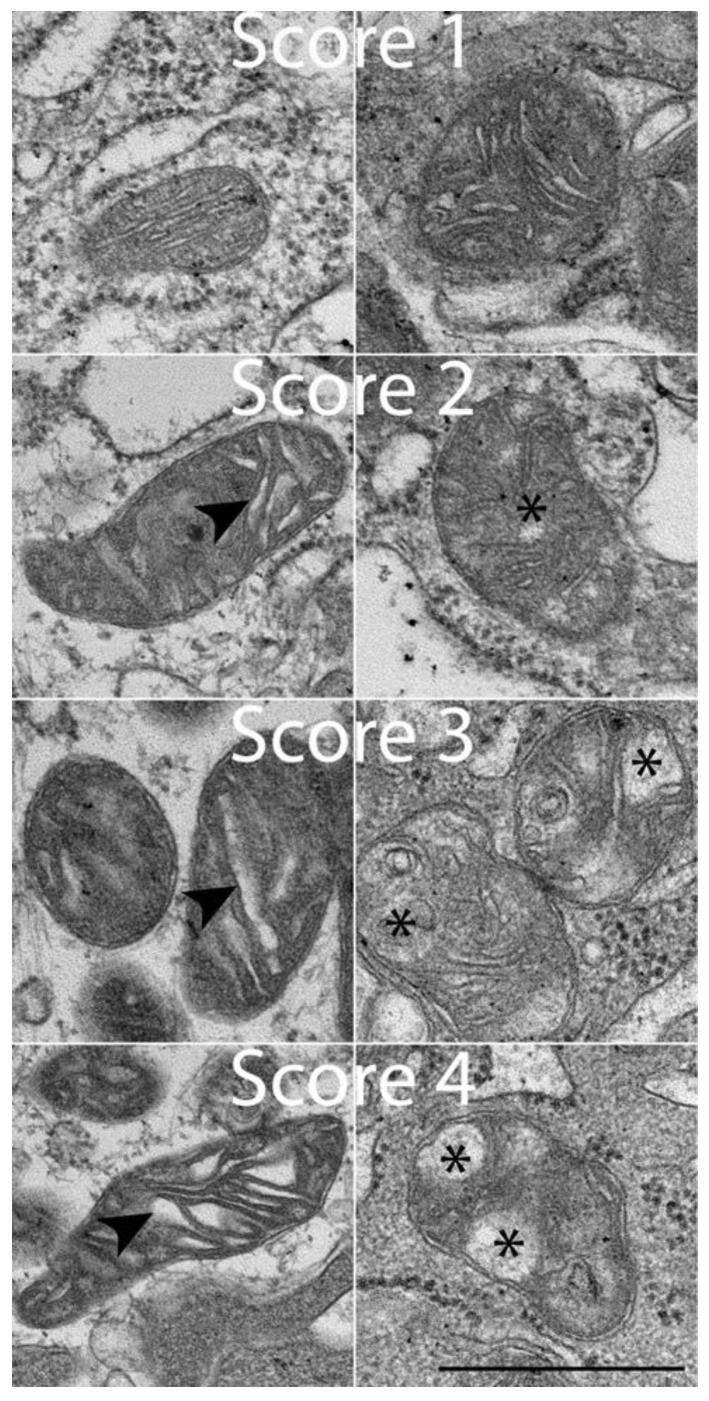
Transmission electron microscopy (TEM) showing the mitochondrial (MITO) scoring of colonocytes. MITO score 1: normal appearance; MITO score 2: some distended cristae (black arrow), slight voids (asterisk); MITO score 3: distended cristae (black arrow) larger voids (asterisks) with some cristae still visible; MITO score 4: major distended cristae (black arrow), large voids (asterisks) with few to no visible cristae.

Qualitative observations made during TEM examination included the assessment of mitochondrial (MITO) lesions and rough endoplasmic reticulum (ER) lesions, scoring the lesions from 1 (normal) to 4 (severe pathology). The qualitative assessment protocol and scoring matrix were developed in-house by T.S. and E.L. specifically for this canine study (Figure 1 and Figure 2; Table 1 and Table 2). Blinding was ensured throughout this study. Colon biopsies were each labeled with consecutive numbers prior to TEM processing and were processed randomly. Image capture and lesion scoring were performed by two microscopists blinded as to treatment visit (pre- vs. post-treatment, i.e., day 0 vs. day 42) and treatment group (SYN vs. PL). Quantitative observations included an assessment of microvillous length (MVL). Ultrastructural changes in MITO, ER, and MVL scores were compared between SYN and PL treatment groups using one-way ANOVA, followed by Tukey’s multiple-comparison test by GraphPad Prism 10 (GraphPad, Boston, MA, USA). A *p* < 0.05 was considered significant.

### 2.5. Lipid Peroxidation Assay

LPx was assessed in serum samples by observing the formation of thiobarbituric acid-reactive substances (TBARSs) [40]. The assay was conducted in the presence of BHT to inhibit any artefactual peroxidation that may occur during the heating process. MDA served as the standard, and TBARSs were quantified in terms of MDA equivalents (nmol/mL). Briefly, the Abcam Lipid Peroxidation (MDA) assay kit was used to estimate malondialdehyde (MDA) concentration in accordance with the manufacturer’s instructions (https://www.abcam.com; Abcam, Waltham, MA, USA). The measurement of MDA was conducted by mixing 20 μL of serum with 500 μL of 42 mM H_2_SO_4_. Subsequently, 125 μL of phosphotungstic acid solution was added, followed by vortexing the mixture, incubation for 5 min, and centrifugation at 13,000× *g* for 3 min. The pellet was resuspended with 100 μL of double-distilled water (ddH_2_O) in addition to 2 μL of butylated hydroxytoluene (BHT) stock (100×), and the final volume was adjusted to 200 μL with ddH_2_O. In each vial, 200 μL of standard or sample was mixed with 600 μL of Developer VII/thiobarbituric acid (TBA) reagent. The mixture was incubated at 95 °C for 60 min and cooled to room temperature in an ice bath for 10 min. Finally, 200 μL of the reaction mixture containing MDA-TBA adduct was transferred into a 96-well plate, and absorbance was recorded at OD 532 using a SpectraMax M2e (Molecular Devices, San Jose, CA, USA) microplate reader. The lipid peroxide level, represented as MDA concentration (nmol/mL), was determined as described by the manufacturer for the kit (https://www.abcam.com; Abcam, Waltham, MA, USA). MDA levels were compared between SYN and PL treatment groups using one-way ANOVA, followed by Tukey’s multiple-comparison test. A *p* < 0.05 was considered statistically significant.

### 2.6. Total RNA Extraction and Sequencing

Canine colon biopsies collected in the RNA*later* Stabilization Solution (ThermoFisher Scientific, San Jose, CA, USA) were preserved at −80 °C until further processing and analysis. RNA extraction, quality assessment, and Illumina sequencing were performed at Novogene Corporation Inc. (Sacramento, CA, USA/Beijing, China). The isolation of total RNA was performed utilizing the TRIzol reagent (Invitrogen, Carlsbad, CA, USA) in accordance with the guidelines provided by the manufacturer. Subsequently, the degradation of RNA and possible contamination were assessed using 1% agarose gels, and the initial quantification of RNA samples was performed with a NanoDrop spectrophotometer (ThermoFisher Scientific, San Jose, CA, USA). The Qubit RNA Assay Kit was utilized with a Qubit 4.0 Fluorometer to determine RNA concentration. The RNA integrity was evaluated with the RNA Nano 6000 Assay Kit using the Agilent Bioanalyzer 2100 system (Agilent Biotechnologies, Santa Clara, CA, USA).

Sequencing libraries were constructed utilizing the NEBNext Ultra II RNA Library Prep Kit for Illumina (New England Biolabs, Ipswich, MA, USA), adhering to the guidelines provided by the manufacturer. Index codes were incorporated to assign sequences to each sample. Initially, mRNAs were isolated from the total RNAs using magnetic beads that were attached to poly-T oligos. Fragmentation was performed utilizing divalent cations at an increased temperature in the NEBNext First Strand Synthesis Reaction Buffer. Subsequently, a random hexamer primer along with M-MuLV Reverse Transcriptase (RNase H-) was employed to synthesize the first strand of cDNA. Subsequently, second-strand cDNA synthesis was conducted utilizing DNA Polymerase I and RNase H. Exonuclease and polymerase activities were employed to transform any remaining overhangs into blunt ends. Following the adenylation of the 3′ ends of the DNA fragments, the NEBNext Adaptor featuring hairpin loop structures was ligated to facilitate hybridization. The AMPure XP system (Beckman Coulter, San Jose, CA, USA) was utilized to select cDNA fragments (150–200 bp). Subsequently, 3 μL of the USER Enzyme (New England Biolabs, Ipswich, MA, USA) with size-selected, adaptor-ligated cDNA were incubated at 37 °C for 15 min, followed by a 5 min incubation at 95 °C, amplification by PCR using Phusion High-Fidelity DNA polymerase (New England Biolabs, Ipswich, MA, USA), and the purification of the PCR products using the AMPure XP System (New England Biolabs, Ipswich, MA, USA). The library’s quality was evaluated using the Qubit 4.0 Fluorometer (Life Technologies, Carlsbad, CA, USA) and real-time PCR for exact quantification, together with the Agilent Bioanalyzer 2100 system for size distribution analysis using the Agilent RNA 6000 Nano Kit (Agilent, Santa Clara, CA, USA). Quantified libraries were combined and sequenced using an Illumina Novaseq6000 platform (Illumina Inc., San Diego, CA, USA), producing 150 bp paired-end reads. The initial processing of raw data (raw reads) in FASTQ format was conducted using Novogene’s in-house Perl scripts. Data were refined by eliminating reads that included adapter sequences, those with poly-N stretches (where N > 10%; N represents an unresolvable base), and reads that contained >50% of low-quality (Qphred <= 5) bases. The clean data underwent calculations for Q20 (99% accuracy), Q30 (100% accuracy), GC-content, and sequence duplication levels. All downstream analyses were conducted using high-quality, clean data.

### 2.7. Transcriptional Analysis of Genes Involved in OS

The reference genome and gene model annotation files for *Canis lupus familiaris* were obtained directly from the genome website [41]. An index of the reference genome was generated, and paired-end clean reads were aligned to the *Canis lupus familiaris* reference genome employing Hisat2 v2.0.5 [42]. Hisat2 was selected as the mapping tool due to its ability to generate a database of splice junctions utilizing the gene model annotation file, resulting in more effective mapping outcomes in comparison to other non-splice mapping tools. FeatureCounts v1.5.0-p3 was employed to count the number of reads aligned to each gene [43]. In RNA-seq, FPKM (expected number of fragments per kilobase of transcript sequence per million base pairs sequenced) takes into account both the sequencing depth and gene length when calculating the read counts concurrently. FPKM is currently the state-of-the-art approach for assessing gene expression levels [44]; hence, we performed FPKM conversion for the read count in the present study.

### 2.8. Analysis of the Enrichment of Differentially Expressed Genes (DEGs)

The analysis of differential expression was conducted for the treatment groups (PL and SYN) through the DESeq2R package (1.20.0) [45,46]. The *p*-values obtained were adjusted using Benjamini and Hochberg’s approach [47]. Genes with adjusted *p* ≤ 0.05, as identified by DESeq2, were classified as DEGs, and the thresholds for significant differential expression were established at log2(fold_change) > 1 [48]. Gene Ontology (GO) enrichment analysis of DEGs was conducted using the clusterProfiler R package (version 4.5; accessed on 5 January 2025), with adjustments made for gene length bias. GO terms with adjusted *p*-values ≤ 0.05 were considered significantly enriched by DEGs. The cluster Profiler R package was used to evaluate the statistical enrichment of DEGs in the Kyoto Encyclopedia of Genes and Genomes (KEGG) pathways [49], and the KOBAS 2.0 web server was used for the annotation and identification of enriched pathways and diseases [50,51]. KEGG serves as a comprehensive resource for interpreting the overarching functions and utilities of biological systems, including cells and organisms, derived from extensive molecular datasets produced by genome sequencing [52].

## 3. Results

### 3.1. Assessment of Ultrastructural Changes

**Figure 3:**
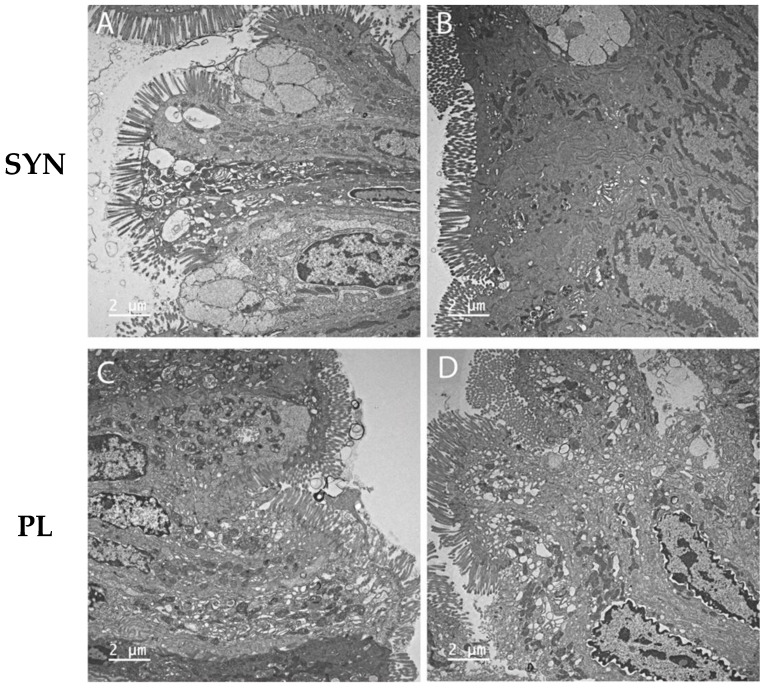
Transmission electron microscopy (TEM) showing comparative effects of treatment on colonocytes. (**A**) CV1SYN—MV separation, large vacuoles (LVs), distended ER, and altered MITO cristae; (**B**) CV3SYN—marked US improvement, MV, ER, MITO appear normal, and no LVs; (**C**) CV1PL—severe changes to MV, ER, and MITO and numerous LV; (**D**) CV3PL—similar or more severe than that seen in (**C**).

The clinical, endoscopic, histopathologic, microbiologic, and local/systemic inflammatory findings of both canine treatment groups have been previously described [9]. The extent of mucosal ultrastructural pathology in the colon varied in individual dogs pre- vs. post-treatment. Pre-treatment ultrastructural changes to colonocytes observed in most dogs included microvilli that were reduced in number, non-uniformly distributed across the luminal surface, and variable in length. Within individual cells, large vacuoles (LVs) were associated with disruption in the cytoplasm, and variably distended ER was present (Figure 1 and Figure 3). Changes in morphology with inflammation included abnormalities to the MITO size and shape, the presence of architectural voids, and distension of cristae (Figure 2). Alterations in the morphology of colonic MVL, MITO, and ER were observed in many dogs, without any significant differences in these ultrastructural parameters identified between PL and SYN group dogs prior to treatment.

**Figure 4:**
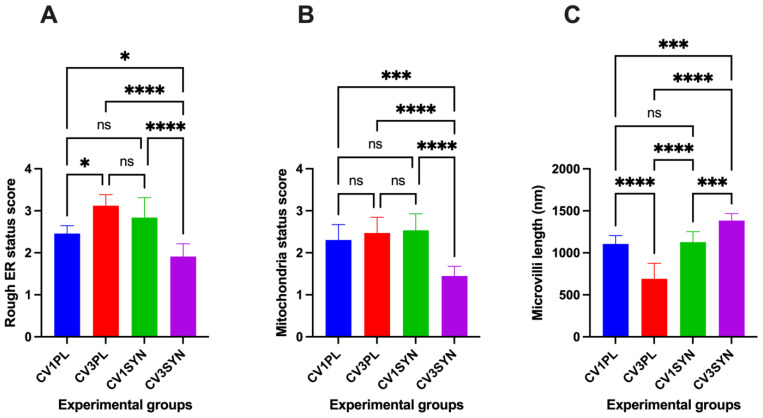
Comparative qualitative assessment of mitochondrial lesions (MITOs) and rough endoplasmic reticulum (ER) lesions between treatment groups (SYN vs. PL). Ultrastructural changes in (**A**) ER, (**B**) MITO scores, and (**C**) microvilli length (MVL) were compared between SYN and PL treatment groups using one-way ANOVA followed by Tukey’s multiple-comparison test. A *p* < 0.05 was considered significant. * Significantly different at *p* < 0.05; *** significantly different at *p* < 0.0005; **** significantly different at *p* < 0.0001; ns = no significant difference. CV1PL = Placebo group client visit 1 (pre-treatment); CV3PL = placebo group client visit 3 (post-treatment); CV1SYN = synbiotic group client visit 1 (pre-treatment); CV3SYN = synbiotic group client visit 3 (post-treatment).

In contrast, significant ultrastructural improvement was observed between the dog groups post-treatment. The SYN-treated dogs exhibited significant (*p* < 0.05) ultrastructural improvements in the MVL, MITO, and ER injury scores when compared to values observed in PL-treated dogs (Figure 4). Specifically, SYN-treated dogs demonstrated superior improvements in cellular architecture characterized by microvilli, which appeared uniform; ER was not distended; large cytoplasmic vacuoles were not present; and MITO appeared normal. In dogs receiving PL, there was no discernible change in the ultrastructural appearance of colonocytes pre- vs. post-treatment (Figure 3).

### 3.2. Assessment of Oxidative Stress Marker

**Figure 5:**
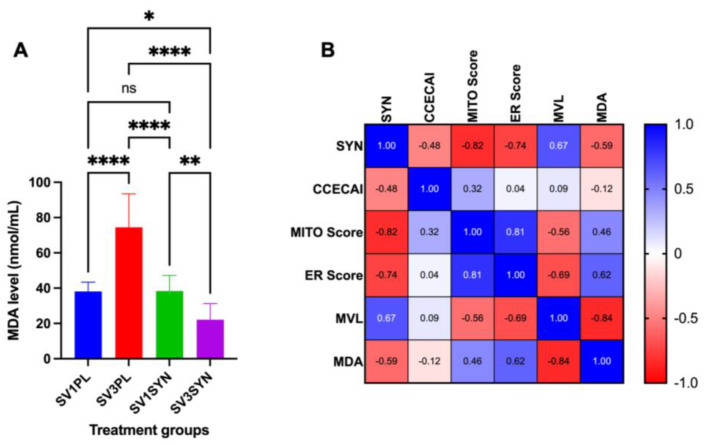
A comparative analysis of oxidative stress and its correlation with ultrastructural changes and disease activity scores in dogs with CE following SYN treatment. (**A**) Comparison of serum MDA levels between treatment groups (SYN vs. PL). * Significantly different at *p* < 0.05. ** Significantly different at *p* < 0.001. **** Significantly different at *p* < 0.0001; ns = no significant difference. SV1PL = Serum from placebo group client visit 1 (pre-treatment); SV3PL = serum from placebo group client visit 3 (post-treatment); SV1SYN = serum from synbiotic group client visit 1 (pre-treatment); SV3SYN = serum from synbiotic group client visit 3 (post-treatment); placebo (PL); synbiotic supplement (SYN). (**B**) A heat map illustrating Pearson correlation coefficients among MDA level, mitochondrial and ER integrity, microvilli length, CCECAI, and synbiotic treatment. Minimal statistical significance was accepted at *p* < 0.05. Positive correlations are represented in blue, while negative correlations are indicated in red. Malondialdehyde (MDA); microvilli length (MVL); serum (S); canine chronic enteropathy clinical activity index (CCECAI); mitochondrial lesion score (MITO score); rough endoplasmic reticulum (ER); lesion score (ER score). Details of the baseline parameters of dogs with CE completing the treatment trial are available in Supplementary Table S1 [9].

The analysis revealed no statistically significant differences in serum MDA concentrations when comparing the SV1PL and SV1SYN groups (Figure 5A). However, serum MDA concentrations were significantly elevated in the SV3PL group when compared to SV1PL (*p*  <  0.0001), SV1SYN (*p*  <  0.0001), and SV3SYN groups (*p*  < 0.0001), respectively. Conversely, the SV3SYN group exhibited the lowest serum MDA concentrations (*p*  <  0.05, Figure 5A). MDA concentrations exhibited a positive correlation with the MITO score (*r* = 0.46; Figure 5B) and ER score (*r* = 0.61; Figure 5B) but showed a negative correlation with SYN treatment (*r* = 0.59; Figure 5B) and MV length (*r* = 0.84; Figure 5B).

### 3.3. Gene Ontology (GO) Functional Annotation of DEGs

**Figure 6:**
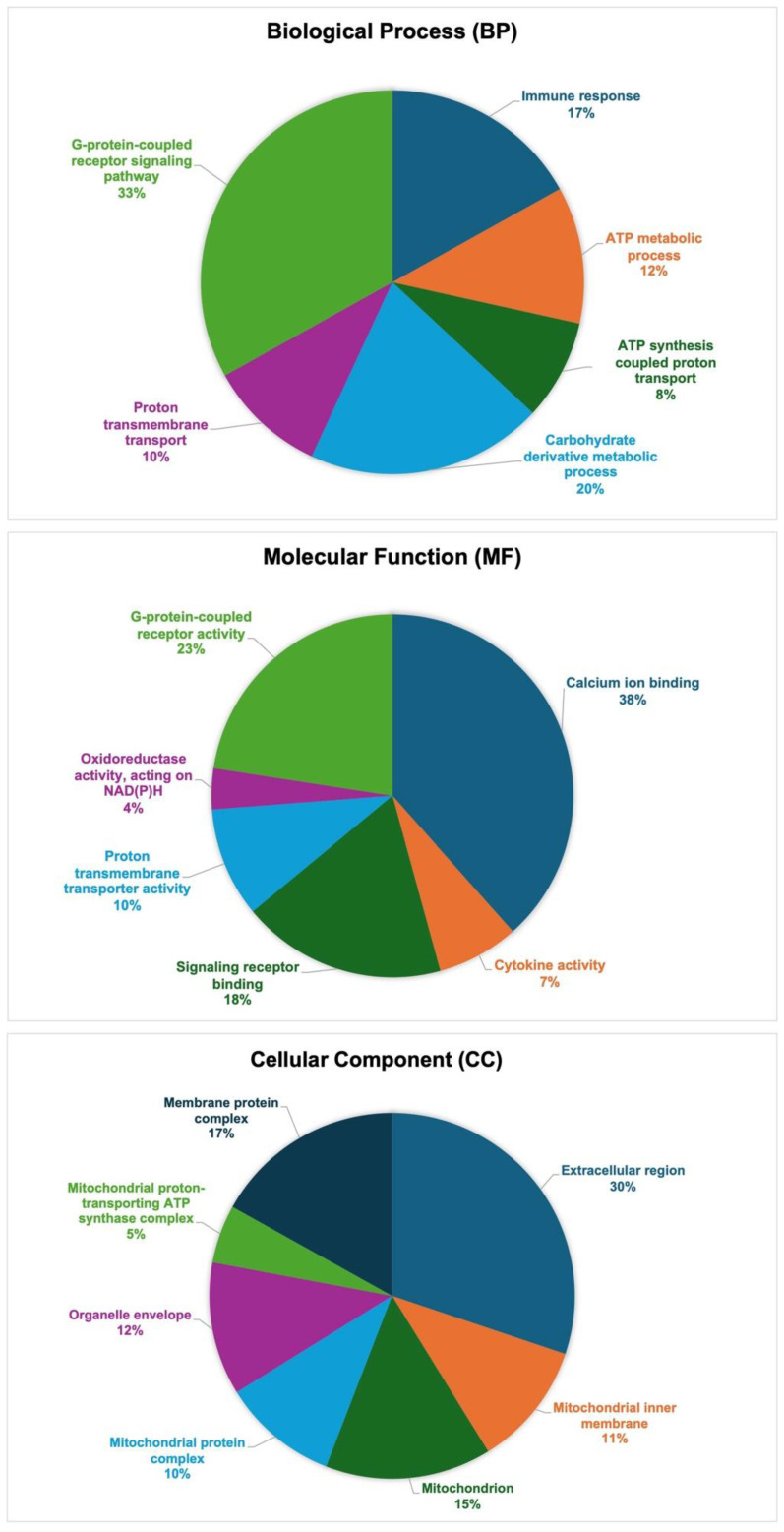
Analysis of Gene Ontology (GO) for differentially expressed genes (DEGs) that are upregulated or downregulated in synbiotic (SYN)-treated dogs. The DEGs are annotated according to biological process (BP), molecular function (MF), and cellular component (CC) categories. The percentages indicate the relative number of genes within each functional category. The DEGs were compared in the colon biopsies of dogs with CIE before (at diagnosis) and after six weeks of SYN treatment.

Differentially expressed genes (DEGs) were compared in the colon biopsies of dogs with CIE before (at diagnosis) and after six weeks of SYN vs. PL treatment. The DEGs (upregulated/UR and downregulated/DR) were organized according to their GO annotations and divided into three primary categories: (i) biological process (BP), (ii) molecular function (MF), and (iii) cellular component (CC). Significantly modulated DEGs following SYN treatment were enriched in (i) the biological process of immune responses (21 UR and 1 DR), ATP metabolic processes (3 UR and 12 DR), ATP synthesis-coupled proton transport (3 UR and 8 DR), carbohydrate derivative metabolic processes (11 UR and 15 DR), proton transmembrane transport (4UR and 9DR), and the G-protein-coupled receptor signaling pathway (41 UR and 2 DR); and in (ii) the molecular function of calcium ion binding (55 UR and 8 DR), cytokine activity (11 UR and 1 DR), signaling receptor binding (29 UR and 1 DR), proton transmembrane transporter activity (5 UR and 11 DR), oxidoreductase activity acting on NAD(P)H (0 UR and 6 DR), and G-protein-coupled receptor activity (35 UR and 2 DR); moreover, (iii) they were predicted to be localized in the extracellular region (37 UR and 4 DR), mitochondrion (3 UR and 17 DR), MITO inner membrane (3 UR and 12 DR), MITO protein complex (3 UR and 11 DR), organelle envelope (3 UR and 13 DR), MITO proton-transporting ATP synthase complex (3 UR and 4 DR), and membrane protein complex (12 UR and 11 DR) (Figure 6).

### 3.4. KEGG Pathway Enrichment Analysis

**Figure 7:**
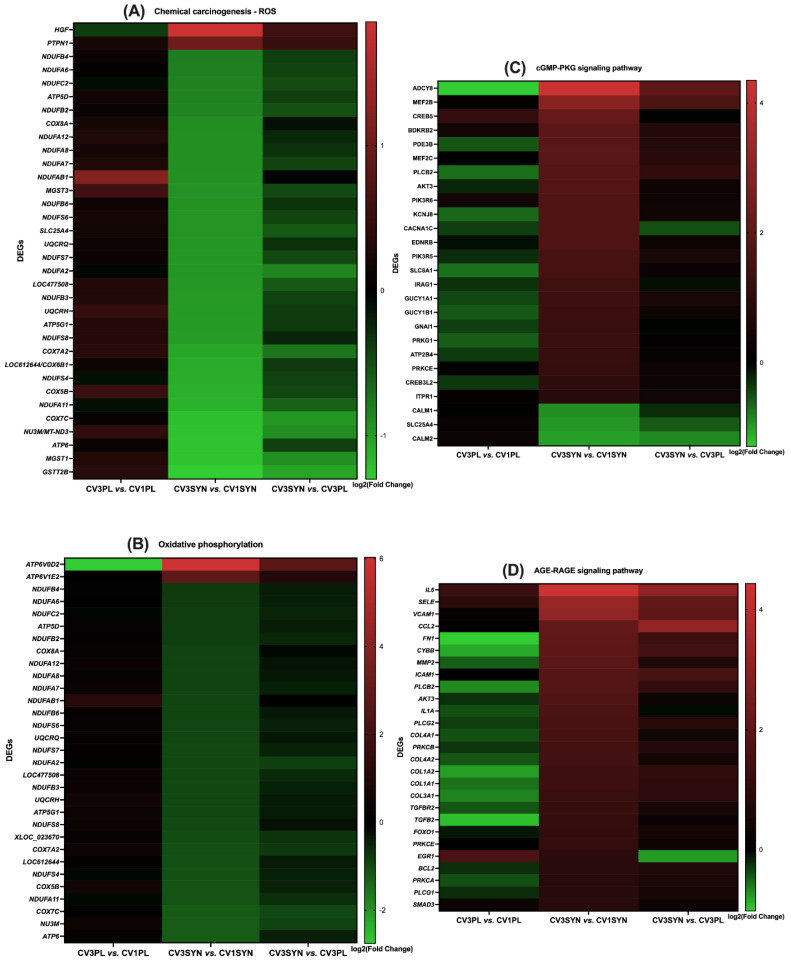
Analysis of heat maps for selected KEGG categories: (**A**) chemical carcinogenesis—ROS; (**B**) oxidative phosphorylation; (**C**) cGMP-PKG signaling pathway; (**D**) AGE-RAGE signaling pathway. Heat maps illustrate color-coded expression levels (log2-fold change) of the most significantly up- (UR) or downregulated (DR) differentially expressed genes (DEGs) in colon tissues of CIE dogs (CV3PL vs. CV1PL, CV3SYN vs. CV1SYN, and CV3SYN vs. CV3PL). More information is available in Supplementary Table S2A–N. CV1SYN: SYN dog group pre-treatment (visit 1); CV3SYN: SYN dog group post-treatment (visit 3); CV1PL: PL group at visit 1 (pre-treatment); CV3PL: PL group at visit 3 (post-treatment); CIE: chronic inflammatory enteropathy; ROS: Reactive oxygen species; cGMP: cyclic guanosine monophosphate; PKG: protein kinase G; AGEs: advanced glycation end products; RAGE: receptor for advanced glycation end products; KEGG: Kyoto Encyclopedia of Genes and Genomes.

**Figure 8:**
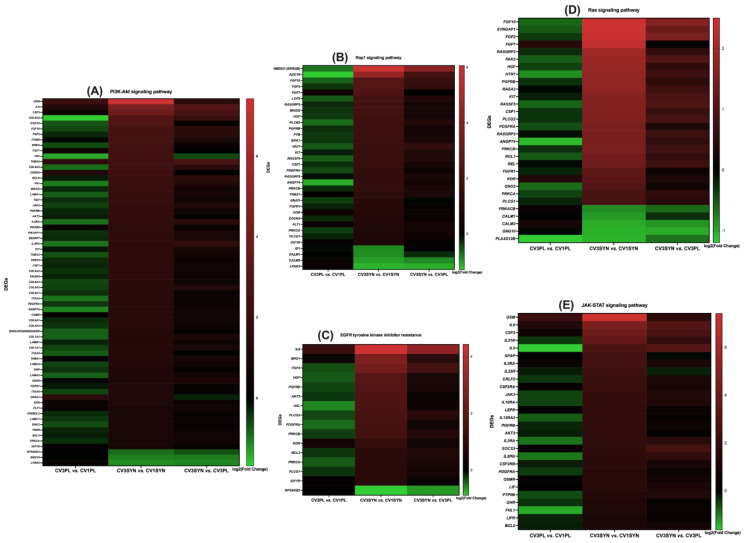
Analysis of heat maps for selected KEGG categories: (**A**) PI3K/Akt, (**B**) Rap1 signaling pathways, (**C**) EGFR tyrosine kinase inhibitor (TKI) resistance, (**D**) Ras, and (**E**) JAK/STAT signaling pathways. Heat maps illustrate color-coded expression levels (log2-fold change) of the most significantly up- (UR) or downregulated (DR) differentially expressed genes (DEGs) in colon tissues of CIE dogs (CV3PL vs. CV1PL, CV3SYN vs. CV1SYN, and CV3SYN vs. CV3PL). More information is available in Supplementary Table S2A–N. CV1SYN: SYN dog group pre-treatment (visit 1); CV3SYN: SYN dog group post-treatment (visit 3); CV1PL: PL group at visit 1 (pre-treatment); CV3PL: PL group at visit 3 (post-treatment); CIE: chronic inflammatory enteropathy; PI3K: phosphatidylinositol-3-kinase; Akt: protein kinase B; JAK: Janus kinase; STAT: signal transducer and activator of transcription; EGFR: epidermal growth factor receptor; KEGG: Kyoto Encyclopedia of Genes and Genomes.

**Figure 9:**
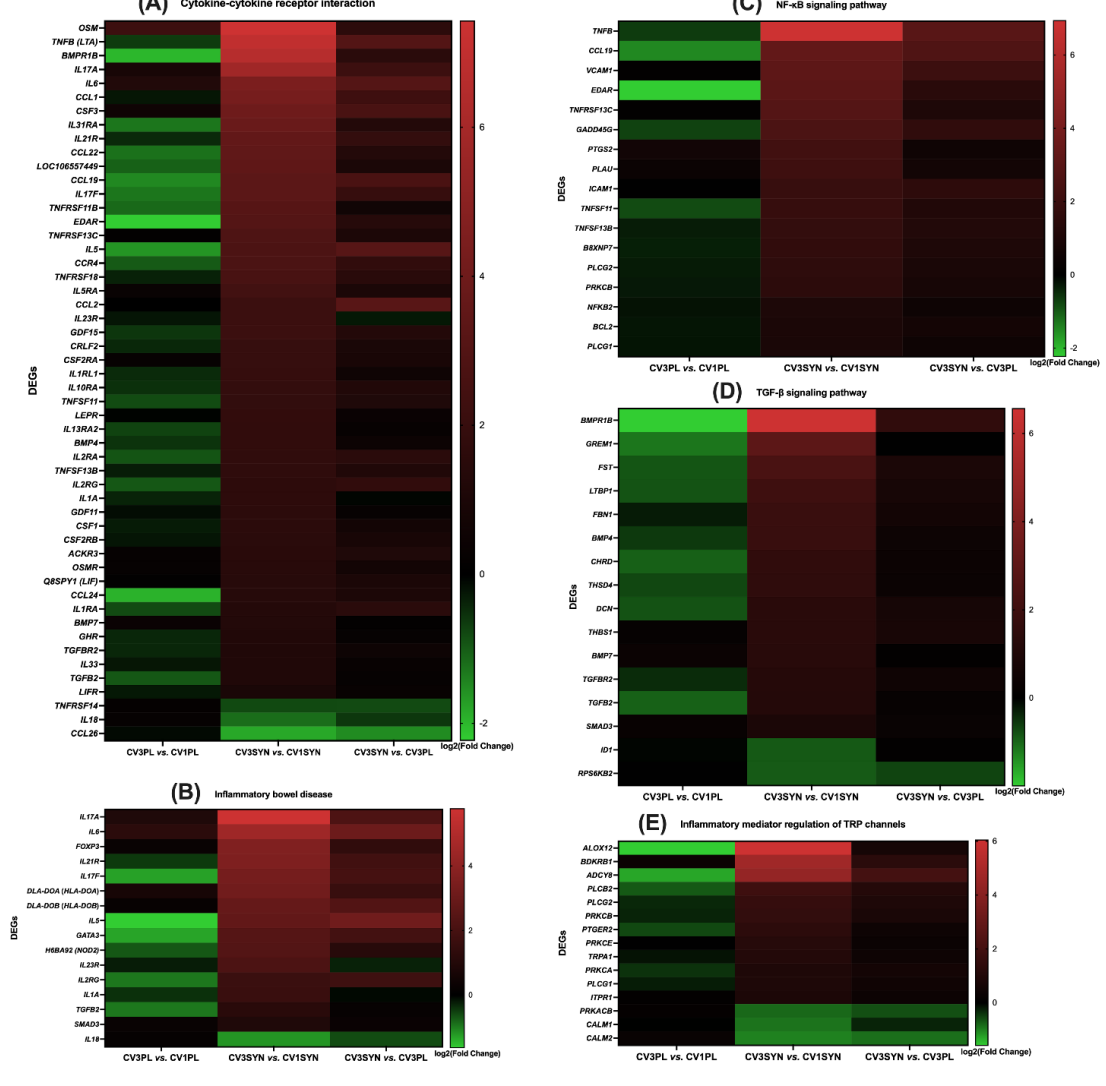
Analysis of heat maps for selected KEGG categories: (**A**) cytokine–cytokine receptor interaction, (**B**) inflammatory bowel disease (IBD), (**C**) NF-κB signaling pathway, (**D**) TGF-β signaling pathways, and (**E**) inflammatory mediator regulation of TRP channels. Heat maps illustrate color-coded expression levels (log2-fold change) of the most significantly up- (UR) or downregulated (DR) differentially expressed genes (DEGs) in colon tissues of CIE dogs. More information is available in Supplementary Table S2A–N. CV1SYN: SYN dog group pre-treatment (visit 1); CV3SYN: SYN dog group post-treatment (visit 3); CV1PL: PL group at visit 1 (pre-treatment); CV3PL: PL group at visit 3 (post-treatment); CIE: chronic inflammatory enteropathy; NF-κB: nuclear factor kappa-light-chain-enhancer of activated B cells; TGF-β: transforming growth factor beta; TRP: transient receptor potential; KEGG: Kyoto Encyclopedia of Genes and Genomes.

The KEGG pathway enrichment analysis provided a more detailed organization and evaluation of the results, grouping associated genes within the same pathway. The phosphatidylinositol-3-kinase/protein kinase B (PI3K/Akt) signaling pathway; oxidative phosphorylation; advanced glycation end products (AGEs) and their receptor (RAGE) (AGE-RAGE) signaling pathway in diabetic complications; chemical carcinogenesis–reactive oxygen species (ROS); Janus kinase/signal transducer and activator of transcription (JAK/STAT) signaling pathway; inflammatory bowel disease; Rap1 signaling pathway; Ras signaling pathway; nuclear factor kappa-light-chain-enhancer of activated B cell (NF-κB) signaling pathway; cyclic guanosine monophosphate (cGMP)–protein kinase G (PKG) (cGMP-PKG) signaling pathway; epidermal growth factor receptor (EGFR) tyrosine kinase inhibitor (TKI) resistance; transforming growth factor beta (TGF-β) signaling pathway; inflammatory mediator regulation of transient receptor potential (TRP) channels; and cytokine–cytokine receptor interaction were identified as the most significantly enriched pathways in KEGG analysis when comparing the colon biopsies of dogs with CIE pre- versus post-SYN treatment (Figure 7, Figure 8 and Figure 9 and Table 3).

**Figure 10:**
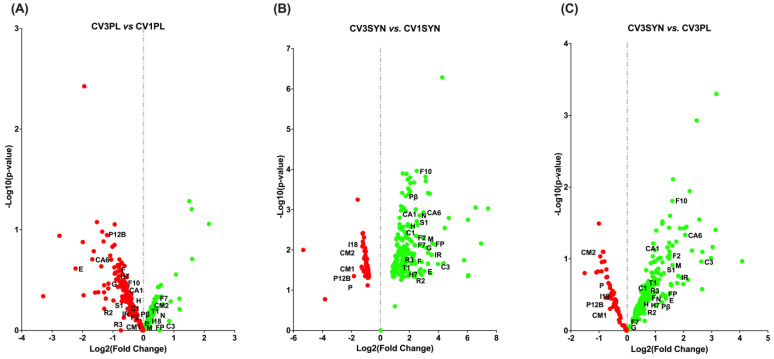
Volcano plots showing gene expression differences between treatment groups CV1 vs. CV3. Differentially expressed genes (DEGs) in the colon mucosa of PL- and SYN-treated groups at visit 1 (pre-treatment; CV1PL and CV1SYN) and visit 3 (post-treatment; CV3PL and CV3SYN): (**A**) CV3PL vs. CV1PL, (**B**) CV3SYN vs. CV1SYN, (**C**) CV3SYN vs. CV3PL. The upregulated DEGs are represented by green dots, and downregulated DEGs are represented by red dots. More information is available in Supplementary Table S3A–N. Calmodulin 1 (*CALM1*; CM1); calmodulin 2 (*CALM2*; CM2); collagen type I alpha 1 chain (*COL1A1*; CA1); colony-stimulating factor 1 (*CSF1*; C1); colony-stimulating factor 3 (*CSF3*; C3); fibroblast growth factor 10 (*FGF10*; F10); fibroblast growth factor 2 (*FGF2*; F2); fibroblast growth factor 7 (*FGF7*; F7); hepatocyte growth factor (*HGF*; H); 5-hydroxytryptamine receptor 7 (*HTR7*; H7); interleukin 21 receptor (*IL21R*; IR); interleukin-18 (*IL18*; I18); platelet-derived growth factor receptor beta (*PDGFR-β*; Pβ); phospholipase A2 group XIIB (*PLA2G12B*; P12B); protein kinase cAMP-activated catalytic subunit beta (*PRKACB*; P); RAS guanyl releasing protein 2 (*RASGRP2*; R2); RAS guanyl releasing protein 3 (*RASGRP3*; R3); synaptic Ras GTPase activating protein 1 (*SynGAP1*; S1); thrombospondin 1 (*THBS1*; T1); myocyte enhancer factor 2B (*MEF2B*; M); forkhead box P3 (*FoxP3*; FP); collagen type VI alpha 6 chain (*COL6A6*; CA6); follistatin (*FST*; F);gremlin 1 (*GREM1*; G); ectodysplasin A receptor (*EDAR*; E); neuregulin 1 (*NRG1*; N).

**Figure 11:**
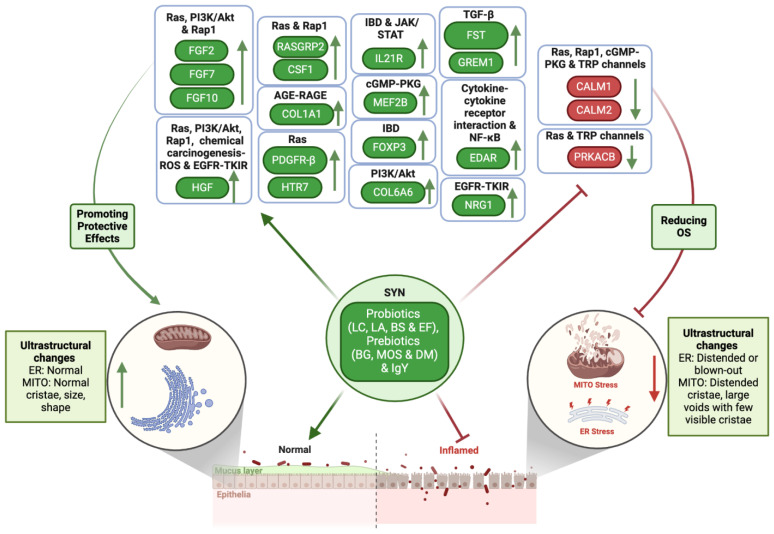
Improvements in ultrastructural changes with the upregulation of protective genes and downregulation of harmful genes following SYN treatment in dogs with CIE. Fibroblast growth factor 2 (*FGF2*). Fibroblast growth factor 7 (*FGF7*). Fibroblast growth factor 10 (*FGF10*). Hepatocyte growth factor (*HGF*). RAS guanyl releasing protein 2 (*RASGRP2*). Colony-stimulating factor 1 (*CSF1*). Collagen type I alpha 1 chain (*COL1A1*). Platelet-derived growth factor receptor beta (*PDGFR-β*). 5-hydroxytryptamine receptor 7 (*HTR7*). Interleukin-21 receptor (*IL21R*). Myocyte enhancer factor 2B (*MEF2B*). Forkhead box P3 (*FoxP3*). Collagen type VI alpha 6 chain (*COL6A6*). Follistatin (*FST*). Gremlin 1 (*GREM1*). Ectodysplasin A receptor (*EDAR*). Neuregulin 1 (*NRG1*). Calmodulin 1 (*CALM1*). Calmodulin 2 (*CALM2*). Protein kinase cAMP-activated catalytic subunit beta (*PRKACB*). Phosphatidylinositol-3-kinase/protein kinase B (PI3K/Akt). Reactive oxygen species (ROS). Epidermal growth factor receptor–tyrosine kinase inhibitor resistance (EGFR-TKIR). Advanced glycation end products and their receptor (AGE-RAGE). Inflammatory bowel disease (IBD). Janus kinase/signal transducer and activator of transcription (JAK/STAT). Transforming growth factor beta (TGF-β). Nuclear factor kappa-light-chain-enhancer of activated B cells (NF-κB). Cyclic guanosine monophosphate–protein kinase G (cGMP-PKG). Inflammatory mediator regulation of transient receptor potential channels (TRP). *Lactobacillus casei* (LC). *L. acidophilus* (LA). *Bacillus* subtilis (BS). *Enterococcus faecium* (EF). Beta-glucans (BG). Mannan oligosaccharides (MOS). D-mannose (DM). Chicken egg yolk immunoglobulin IgY (IgY). This figure was created in BioRender (Sahoo, D. (2025) https://BioRender.com/l28f579; accessed on 10 February 2025). Upward-pointing arrows signify an increase, while downward-pointing arrows indicate a decrease.

For CV3SYN (colon tissues post-SYN treatment) compared to CV1SYN (colon tissues pre-SYN treatment), a number of genes associated with protective effects, including fibroblast growth factor 2 (*FGF2*), fibroblast growth factor 7 (*FGF7*), fibroblast growth factor 10 (*FGF10*), synaptic Ras GTPase activating protein 1 (*SynGAP1*), RAS guanyl releasing protein 2 (*RASGRP2*), RAS guanyl releasing protein 3 (*RASGRP3*), thrombospondin 1 (*THBS1*), colony-stimulating factor 1 (*CSF1*), colony-stimulating factor 3 (*CSF3*), interleukin 21 receptor (*IL21R*), collagen type I alpha 1 chain (*COL1A1*), hepatocyte growth factor (*HGF*), 5-hydroxytryptamine receptor 7 (*HTR7*), platelet derived growth factor receptor beta (*PDGFR-β*/*PGFRB*), collagen type VI alpha 6 chain (*COL6A6*), ectodysplasin A receptor (*EDAR*), forkhead box P3 (*FoxP3*), follistatin (*FST*), gremlin 1 (*GREM1*), myocyte enhancer factor 2B (*MEF2B*), and neuregulin 1 (*NRG1*), were upregulated (Figure 10 and Figure 11, Table 3, and Supplementary Figure S1). However, several genes exhibiting adverse effects, including adenylate cyclase 8 (*ADCY8*); transforming growth factor-β1 (*TGF-β1*); bradykinin B1 receptor (*BDKRB1*); and bone morphogenetic protein receptor type 1B (*BMPR1B*), along with *IL-31*, *integrin*, *E-selectin*, TNF receptor superfamily member 13C (*TNFrsf13C*), vascular cell adhesion molecule-1 (*VCAM-1*), tumor necrosis factor (*TNF*), and lymphotoxin (*LT*), were also upregulated following SYN treatment. Conversely, several other genes linked to adverse effects, such as protein kinase cAMP-activated catalytic subunit beta (*PRKACB*), phospholipase A2 group XIIB (*PLA2G12B*), calmodulin 1 (*CALM1*), calmodulin 2 (*CALM2*), and interleukin-18 (*IL18*), were significantly downregulated (Figure 10 and Figure 11, Table 3, and Supplementary Figure S1). The observed DEG changes in CV3SYN vs. CV1SYN were similar to those observed for CV3SYN vs. CV3PL, highlighting a significant upregulation of protective genes, including *FGF2*, *FGF10*, *SynGAP1*, *CSF3*, *COL6A6*, and IL21R in SYN-treated dogs. The genes, including *PRKACB*, *PLA2G12B*, *CALM1*, and *CALM2*, associated with negative effects were downregulated in SYN group dogs post-treatment (Figure 10 and Supplementary Figure S1). In PL group dogs (CV3PL vs. CV1PL), genes associated with negative outcomes, such as *PRKACB*, *CALM2*, and *IL18*, were observed to be upregulated, though they were not statistically significant, while genes linked to positive outcomes, such as *FGF2*, *FGF10*, *SynGAP1*, *RASGRP2*, *RASGRP3*, *IL21R*, *COL1A1*, *HGF*, *HTR7*, *PGFRB*, *COL6A6*, *EDAR*, *FoxP3*, *FST*, *GREM1*, *MEF2B*, and *NRG1*, were downregulated (Figure 10 and Supplementary Figure S1).

The protective genes that showed increased expression in the SYN group encompassed *COL1A1* associated with the AGE-RAGE signaling pathways; *FGF2*, *FGF7*, and *FGF10* linked to the Ras, PI3K/Akt, and Rap1 signaling pathways; *RASGRP2* and *CSF1* from the Ras and Rap1 signaling pathways; *HGF* involved in Ras, PI3K/Akt, Rap1, and chemical carcinogenesis–ROS signaling pathways; EGFR-TKI resistance and *IL21R* from the IBD and JAK/STAT signaling pathways; *PDGFR-β* and *HTR7* from the Ras signaling pathway; *COL6A6* from PI3K/Akt; *FOXP3* from IBD; *FST* and *GREM1* from TGF-β; *EDAR* from cytokine–cytokine receptor interactions and NF-κB; *MEF2B* from cGMP-PKG; and *NRG1* from EGFR-TKIR signaling pathways. Additionally, within the SYN group, the genes that were downregulated and had negative implications comprising *CALM1* and *CALM2* from the TRP channels, as well as from the Ras, Rap1, and cGMP-PKG signaling pathways, including *PRKACB* from the Ras signaling pathway and TRP channels (Figure 7, Figure 8 and Figure 9 and Table 3).

## 4. Discussion

The development of chronic diseases is linked to the overproduction of inflammatory cytokines and factors that induce OS [40,53,54,55,56,57,58,59,60,61,62,63,64]. In studies of humans and animal models, acute and chronic GI disorders, including gastroduodenal ulcers, GI cancer, and IBD, are marked by increases in reactive oxygen species (ROS) and the reduced production of antioxidant defenses, both of which disturb redox balances [65]. A consequence of these perturbations is damage to cellular components, the activation of pro-inflammatory signaling pathways, and the dysfunction of the intestinal epithelial barrier [65,66]. Considering the differential effects of probiotics and prebiotics, whether complementary or synergistic, there is increasing evidence indicating that they possess antioxidant activities in humans and animal species [30].

In the current study, a novel synbiotic (Intesto-Guard™) containing probiotic strains *Lactobacillus casei*, *L. acidophilus*, *Bacillus subtilis*, and *Enterococcus faecium*; prebiotics (beta-glucans, mannan oligosaccharides, and D-mannose); and chicken egg yolk immunoglobulin IgY was administered daily to randomized CIE dogs for 42 days [9]. Colon biopsies from pet-owned dogs with CIE were used for analyses in this current study. Our clinical trial results indicate that synbiotic-treated dogs, compared to placebo group dogs, have favorable changes in their mucosal microbiota despite both treatment groups having similar lifestyles, environmental exposures, and the same dietary restrictions [9]. Besides the action of the synbiotic, other factors, including stress, might influence intestinal bacterial populations through the activation of the hypothalamic–pituitary axis (HPA) and endogenous glucocorticoid secretion. Studies in different rodent models have shown that acute stress can modulate the normal intestinal microbiota [67,68,69]. For example, increased levels of corticosterone are observed in germ-free mice undergoing restraint procedures [70]. In another study, neonatal rats administered probiotics shortly after birth are protected against harmful HPA responses and intestinal barrier dysfunction [71]. Stress also increases corticosteroid levels to modulate the murine microbiota by decreasing levels of beneficial *lactobacilli* and increasing the levels of phlogistic *E. coli*, *Pseudomonas* [72,73], and bacterial virulence genes to negatively affect intestinal function [72]. Finally, the role of stress has been investigated in a large animal model, showing that prebiotic administration can reduce the inflammatory response, fecal dysbiosis, and perturbed metabolome in laboratory-reared beagles [74].

Dogs with CIE treated with synbiotic demonstrated significantly improved colonic ultrastructure compared to dogs treated with a placebo. All of the components in SYN [9] exhibit some antioxidant and anti-inflammatory functions, alongside reducing MITO and ER stress and apoptosis, to support GI epithelial barrier integrity. For instance, several studies indicate that IgY may diminish apoptosis in intestinal epithelial cells, reduce caspase activity, and alleviate OS and inflammatory responses in the intestine. Furthermore, IgY enhances the activities of antioxidant enzymes, thereby mitigating tissue damage and preserving the integrity of tissue structure [75,76]. D-mannose, a natural bioactive monosaccharide, has been shown to reduce OS and increase Treg cell proportions in mice with UC [77], demonstrating notable anti-inflammatory and antioxidative properties and thus could be responsible for synergistic effects for improved MITO integrity, as shown in the present study. Mannose also rescues autophagy flux and reverses the senescence-associated secretory phenotype (SASP) [78], blocks phagocytosis, reduces the pro-inflammatory response, and enhances the anti-inflammatory activity of macrophages [79]. Moreover, the administration of mannose mitigates intestinal barrier impairment in murine colitis models and improves lysosomal integrity, thereby averting MITO dysfunction [79]. As lysosomal cathepsin B plays an important role in causing MITO damage in colonic epithelial cells, the protection offered by mannose against mitochondrial dysfunction is linked to its ability to inhibit the release of cathepsin B [80]. While the integrity of the intestinal epithelium and the preservation of tight junctions are key cellular mechanisms reliant on proper MITO functioning, the administration of mannose to the impaired colonic epithelium enhances MITO functioning, thereby supporting the energy production necessary for the expression of tight junction proteins [80]. In contrast to treatment with DSS alone, the combination of DSS and mannose led to significant improvements in mitochondrial function, evidenced by an increase in mitochondrial mass, lower levels of mitochondrial oxidants, and enhanced mitochondrial membrane potential. Furthermore, mannose treatment significantly reinstated the expression of respiratory complexes I (NADH-ubiquinone oxidoreductase), II (succinate dehydrogenase), III (ubiquinol–cytochrome c oxidoreductase), IV (cytochrome c oxidase), and V (ATP synthase) in cells subjected to DSS treatment [80]. D-mannose can compete with glucose (for the same transporter) and hexokinase [81]. Such competition inhibits glycolysis and diminishes mitochondrial ROS. Also, the coculture of murine DSS-treated colon cells with mannose results in notable enhancements in both basal and maximum MITO respiration [80].

Similarly, mannan oligosaccharides (MOS) may diminish the activity of inflammatory genes and lower the production of inflammatory cytokines [82] and ROS while enhancing the levels of Nrf2, antioxidant enzymes, and molecules [83]. The addition of MOS was also found to reduce apoptosis by blocking the death receptor pathway and MITO pathway processes [83]. Similarly, beta-glucan may function as an antioxidant targeted at mitochondria [84]. Beta-glucan pre-treatment demonstrated a reduction in mitochondrial ROS formation, OS, glutathione oxidation, and MITO swelling. It also protects against MITO outer membrane damage and the release of cytochrome c from mitochondria, while inhibiting the decline in ATP production [84] and boosting MITO content and membrane potential [85].

Genes and proteins that confer resistance to OS are essential for the redox mechanisms in *Lactobacillus* spp. For instance, the role of thioredoxin, thioredoxin reductase, and their encoding genes in combating OS (collectively referred to as the thioredoxin antioxidant system) and the importance of Nrf-2 in the redox function of *Lactobacillus* spp. have been emphasized in multiple laboratory studies [86,87,88]. The redox mechanism involves *L. casei* Shirota, wherein the modulation of Nrf-2/Keap 1 signaling and the inhibition of the NF-κB inflammatory pathway play significant roles in the protective effect in relation to the regulation of p65 phosphorylation and GPx2 activity [88]. The cell-free extract of *Lactobacillus* spp. (CFEL) or treatment with *Lactobacillus* spp. enhances the MITO membrane’s potential and MITO contents, which contribute to MITO respiration. This occurs by increasing the expression of respiratory complex subunits, thus providing protection against MITO dysfunction through MITO biogenesis and reducing ROS production and ER stress [89,90]. Moreover, *Lactobacillus* spp. were reported to prevent the LPS-induced disruption of MITO morphology, including cristae structures [89]. Evidence indicates that certain metabolites, such as fatty acids and amino acids, enhance the antioxidative capacity of CFEL, facilitating the removal of ROS [91]. CFEL reduces inflammatory responses by lowering IL-1β levels and downregulating NF-κB expression [90]. *L. casei* Shirota has also been shown to prevent membrane barrier disruption [88], and *Lactobacillus* spp.-derived exopolysaccharide (EPS) supplementation reduces OS by altering gut microbiota composition [92]. Similarly, the pre-treatment of cells with *Bacillus subtilis* spores maintains normal levels of intracellular ROS and GSH while inhibiting the activation of the MAPK cascade, positively influencing cell proliferation [93]. The spores of *B. subtilis* demonstrate their protective mechanism through the nuclear translocation of Nrf-2, which plays a crucial role in activating genes associated with stress responses [93]. The dietary administration of *B. subtilis* has been shown to reduce gut inflammation and enhance antioxidative status and barrier integrity in the duodenum by modulating the gut microbial composition [94]. Likewise, *Enterococcus faecium* demonstrated a protective effect against gastric injury in rats, as indicated by a reduction in ulcer index, histological lesions, gastric pH, and mucosal inflammatory responses, along with increased mucosal glycoprotein production and anti-oxidative function [95].

MDA is an organic compound that arises naturally as a result of lipid peroxidation and is recognized as a significant biomarker for OS [96]. MDA levels are frequently evaluated to determine the efficacy of antioxidant treatments [65,97]. A reduction in MDA levels following treatment may suggest that the therapy effectively diminishes OS [65,97]. The present study further supported this finding, demonstrating a notable reduction in MDA levels in the serum of dogs with CIE after SYN treatment in comparison to PL treatment. In addition to various immunoregulatory factors, ROS are generated at increased levels in IBD. This includes MDA, which has been shown to be generated in IBD patients, and MDA levels correlate with the severity of the disease [65,66]. Dogs experiencing acute diarrhea exhibited increased levels of ROS and OS index values when compared to control indices [98]. Various metabolomic studies in dogs with CIE also reveal alterations in serum and fecal metabolites, which are indicative of significant OS at the time of diagnosis [98].

Electron microscopy (EM) can provide high-resolution analysis of tissues, complementing histopathologic results using light microscopy. Several studies have used EM to evaluate the ultrastructure of inflamed intestinal mucosa with chronic gastroenteritis. Significant subcellular morphological changes have been observed with both CD [15,17,18,19,20,21,22,23,24,99] and UC [15,20,21,25,26,27], with Aluwihare [17] first reporting changes in the colonic ultrastructure of early and established CD compared to normal colons. The relevant findings included the dense infiltration of immune cells in the lamina propria (LP), epithelioid cell granulomas in the submucosa, and the presence of bacteria deep in the LP and submucosa in some patients. Several other studies have used SEM in adult patients with CD [14,17,20,21,22,23,24,99] or UC [25,26,27]. Dvorak et al. performed a detailed SEM study of the ileum and colon in CD, demonstrating changes in villous morphology, goblet cell alterations, and increased mucus secretion [99]. Other studies have suggested that SEM examination could aid in differentiating UC from CD in adults [21] and children [15]. Marin et al. proposed a model for the pathophysiology of CD using a combination of SEM, TEM, and freeze–fracture techniques [19]. The increased prevalence of ultrastructural abnormalities in the upper small intestine of patients with CD has also been described [23]. Shields et al. performed a detailed SEM morphometric analysis demonstrating how SEM was a useful adjunct to light microscopy for the diagnosis of colonic dysplasia [26]. Several other studies have defined the early morphological lesions in CD [14,24] and the ultrastructural features of villous atrophy seen with gluten-sensitive enteropathy [100,101].

Ultrastructural changes to colonocytes have also been observed in the rodent models of IBD. In one study, both TEM and SEM were used to investigate the effects of 2,4,6-trinitrobenzene sulfonic acid (TNBS) colonic infusion, which induces colitis in rats [102]. Ultrastructural changes in colonic epithelial cells included the depletion of goblet cell mucin, alterations to the size and shape of the Golgi apparatus, and the appearance of small vesicular (ER-like) structures in the apical region of colonocytes. Whether these ultrastructural changes induced by TNBS infusion were associated with an IBD-like inflammatory process or the result of TNBS cytotoxicity, however, could not be determined. In another study, the TNBS-colitis model was used to investigate the ultrastructural changes in inflamed and non-inflamed intestines in rats [103]. The results from SEM showed deformations in the mitochondria and Golgi apparatus and the fragmentation of the ER in both the ileal (non-inflamed) and colonic (inflamed) segments.

The present study is the first conducted on dogs with CIE treated with a synbiotic and revealed striking differences in colonic ultrastructural variables in response to SYN treatment. While both treatment groups had comparable ultrastructural pathology at diagnosis, only SYN-treated dogs showed significant improvements in ER and MITO ultrastructure injury scores and MVL compared to their pre-treatment values and also compared to PL. Our ultrastructural assessment of colonocyte pathology using both quantitative and qualitative variables involved the examination of MITO size and shape; cytoplasmic vacuolation; microvillous abundance, length, and vesiculation; and intercellular (tight junction) integrity [20]. We examined all these parameters using an in-house derived measuring and scoring protocol that provided a consistent assessment of ultrastructural changes in colon tissues. The findings of ultrastructural changes to inflamed canine colonocyte microvilli, mitochondria, and ER align with those described in adult CD [14,17,20,23,24,99] and UC [25,26,27] and rodent models of experimental colitis [102,103].

Walker et al. provide the only other description of intestinal ultrastructural lesions in dogs with the food-responsive phenotype of CIE [28]. In this study, endoscopic biopsies of the duodenum revealed significant MITO lesions (cristolysis and swelling), cytoplasmic vacuolation, and abnormalities to the brush border (increased intermicrovillar space) before dietary therapy. Following six weeks of dietary intervention with a hydrolyzed protein (soy-based) diet, dogs showed clinical and laboratory remission accompanied by improved duodenal ultrastructural lesions (reduced MITO lesion scores, decreased intermicrovillar space, and increased microvillous height). Cytoplasmic vacuolation in colonocytes, observed in food-responsive dogs at presentation but failed to improve with diet intervention, was frequently observed in the dogs of the current study. Finally, gluten-sensitive Irish Setters develop early-onset ultrastructural lesions in the jejunum characterized by microvilli that are stunted and reduced in number and by a reduced or absent glycocalyx [104].

The present investigation also elucidated the possible molecular mechanisms through which SYN treatment mitigates OS and enhances MITO and ER health by preventing the progression of OS and inflammation associated with CIE. To explore the signaling pathways that contribute to the reduction in MITO and ER stress, we performed a transcriptomic analysis revealing multiple activated signaling pathways and key genes. The genes that are differentially expressed in the colon due to SYN are primarily enriched in various signaling pathways, including PI3K/Akt, oxidative phosphorylation, AGE-RAGE, chemical carcinogenesis–ROS, JAK/STAT, inflammatory bowel disease, Rap1, Ras, NF-κB, cGMP-PKG, EGFR-TKI resistance, TGF-β, inflammatory mediator regulation of TRP channels, and cytokine–cytokine receptor interaction. The augmented expression of genes associated with the maintenance of MITO and ER ultrastructural integrity, as well as the reduction in OS, such as *FGF2*, *FGF7*, *FGF10*, *SynGAP1*, *RASGRP2*, *RASGRP3*, *THBS1*, *CSF1*, *CSF3*, *IL21R*, *COL6A6*, *EDAR*, *FoxP3*, *FST*, *GREM1*, *MEF2B*, *NRG1*, *COL1A1*, *HGF*, *HTR7*, and *PDGFR-β*, occurred concurrently with the downregulation of genes associated with adverse effects, including *PRKACB*, *PLA2G12B*, *CALM1*, and *CALM2*, following the SYN treatment. However, with the PL treatment, there was a minor upregulation of *PRKACB* and *CALM2* genes, which are associated with adverse effects on the host. Also, several other genes linked to protective effects (*FGF2*, *FGF10*, *SynGAP1*, *RASGRP2*, *RASGRP3*, *COL6A6*, *EDAR*, and *IL21R*) were downregulated.

In contrast, several genes demonstrating negative impacts, such as *ADCY8*, *TGF-β1*, *BDKRB1*, *BMPR1B*, *IL-31*, *integrin*, *E-selectin*, *TNFrsf13C*, *VCAM-1*, *TNF*, and *LT*, were found to be upregulated after SYN treatment. The following all point towards their adverse effects: the overexpression of *ADCY8* associated with the activation of signaling pathways related to inflammation [105]; Atp6v1e2 small hairpin RNAs (shRNA) attenuating the clustering of (ROS)-producing Nox4 [106]; pro-inflammatory cytokine interleukin 31 (IL-31) and its heterodimeric receptor composed of IL-31RA and oncostatin M receptor (OSMR); BDKRB1 contribution to pro-inflammatory chemokine IL-8 production [107] and IL-8 production in OS [108]; association of the genetic variant BMPR1B related to oxidative signaling pathways and clinical complications [109]; TGF-β1 induced ROS production [109]; OS and DNA damage response accompanied by increased Grin2b [110]; increased expression of E-selectin by pro-inflammatory cytokines and OS [111]; inhibited *TNFrsf13C* gene expression, reducing inflammation in RAW 264.7 cells [112]; ROS generation by integrins through cyclo-oxygenase-2 and mitochondria and also by synergistically participating in the crosstalk with EGFR [113]; regulation of *VCAM-1* gene expression coupled to OS; and the role of SOD expression in suppressing the TNF-α-induced expression of VCAM-1 [114].

Conversely, the increased levels of IL-17A and IL-17F following SYN treatment may confer a protective benefit. The interleukin 17 (IL-17) family comprises six structurally related cytokines, ranging from IL-17A to IL-17F [115]. Though interleukin 17A (IL-17A, commonly known as IL-17) has garnered significant interest due to its pro-inflammatory role [115], it plays varying roles depending on the tissue, contributing to health during responses to injury, physiological stress, and infection. The production of IL-17 is increased in cases of IBD [116], but healthy intestines also harbor notable populations of IL-17-producing cells. The IL-17 driven by microbiota influences the local epithelium to enhance anti-microbial responses, which are essential and adequate for sustaining a homeostatic balance while avoiding significant inflammation in the healthy gut [117,118]. Clinical trials investigating neutralizing antibodies against IL-17 and IL-17-binding receptor (IL-17RA) in patients with Crohn’s disease revealed unexpectedly modest effectiveness. Notably, disease exacerbations were reported in some patients treated with secukinumab (anti-IL-17) [119], and increased serum C-reactive protein concentrations, a marker of inflammation, were reported in patients treated with brodalumab (anti-IL-17RA) [120]. Murine studies also demonstrated that the absence of IL-17 signaling can worsen colitis-associated epithelial injury and intestinal leakage. These findings indicate that IL-17 plays a beneficial role in the intestinal epithelium by aiding in the maintenance of the epithelial tight-junction barrier during inflammatory conditions [121,122,123]. IL-17F has the greatest resemblance to IL-17 regarding its cellular origins and functional roles. IL-17F plays a significant role in inflammatory responses and offers protection at barrier surfaces, as demonstrated by the increased susceptibility to chronic mucocutaneous candidiasis in individuals with an autosomal dominant deficiency of *IL-17F* [124].

SYN treatment was ineffective in decreasing CIE-induced gene expression related to TRP channels and NF-kB signaling pathways. The TRP cation channels respond to various exogenous and endogenous biomolecules, with aldehydes identified as a trigger for TRP channels contributing to disease pathophysiology [125]. In reaction to lipid peroxidation caused by inflammation, polyunsaturated fatty acids are converted into aldehydes, including 4-hydroxynonenal. Reactive aldehydes stimulate TRP channels through the formation of protein adducts that release pro-inflammatory mediators, promoting cellular injury and pain [125]. Both TRP cation channels signaling pathway genes, arachidonic acid 12-lipoxygenase (*ALOX12*), and *BDKRB1* (bradykinin receptor B1) expression showed an increase with SYN treatment. ALOX12 can enhance the activity of NADPH oxidase, leading to the production of ROS, such as superoxide and hydrogen peroxide. Furthermore, ALOX12 is also capable of inhibiting the nuclear accumulation of the Nrf2 antioxidant gene activator [126]. It was reported that antagonism of bradykinin B1 can inhibit OS [127]. However, several other genes with adverse effects, including *CALM1* and *CALM2* linked to TRP channels; Ras, Rap1, and cGMP-PKG signaling pathways; and *PRKACB*, were downregulated following SYN treatment. Protein kinase A (PKA) detects OS through redox modifications in its catalytic β subunit (PRKACB) at Cys200 and Cys344, illustrating its function as a typical redox sensor [128]. The activity of PRKACB can be inhibited through treatment with the antioxidant N-acetyl cysteine (NAC) [128]. Similarly, SYN treatment also suppressed *PRKACB* by lowering ROS levels, as evidenced by the reduced expression of genes associated with the oxidative phosphorylation signaling pathway. The presence of OS leads to increased concentrations of intracellular calcium, which influences the MITO and nuclei, resulting in excitotoxicity. As a result, there is an increase in the expression of calmodulin in response to OS [129]. Thus, the decrease in *CALM1* and *CALM2* expression may indicate lowered OS following SYN treatment.

The relationship between the redox status of mucosal glutathione and inflammation, as well as disease progression, suggests that compromised mucosal antioxidant defenses play a significant role in the onset of human UC [130]. The chronic activation of NF-ĸB leads to cellular infiltration and mucosal inflammation by enhancing the transcription of pro-inflammatory cytokines. This process also contributes to the degradation of the intestinal barrier through the increased apoptosis of intestinal epithelial cells [65] and the release of ROS metabolites, which activate NF-ĸB to compromise intestinal barrier stability [131].

While some genes associated with oxidative phosphorylation showed decreased expression following SYN treatment, the gene *Atp6v0d2* showed increased expression after SYN treatment. Reports indicate that *Atp6v0d2*-deficient macrophages exhibit increased MITO damage, suggesting that *Atp6v0d2* plays a role in preserving MITO integrity [132]. Moreover, G-protein subunit gamma 10 (*GNG10*) and phospholipase A2 group XIIB (*PLA2G12B*), associated with the Ras signaling pathway, were downregulated following SYN treatment. GNG10, belonging to the γ subunit family of heteromeric G-protein, is more likely to elicit negative effects (like in the present study) similar to those previously reported for GNG11 [133]. GNG11 is noted for its rapid activation by agents that trigger senescence, such as H_2_O_2_. Moreover, the overexpression of *GNG11* leads to the activation of ERK1/2 of the MAPK family while not affecting Ras [133]. Phospholipase A2 group XIIB (PLA2G12B) is calcium-dependent and is closely connected with the ER membrane [134]. While studies on the molecular mechanisms underlying atherosclerosis suggest increased OS, production of AGEs, and chronic inflammation [135], *PLA2G12B*-mutant mice are atherosclerosis-resistant [134]. The present study suggests that the reduction in *PLA2G12B* expression could be associated with decreased levels of OS, which are marked by reduced MDA levels following SYN treatment. Increased ROS could also be due to AGE-RAGE-oxidative stress (AROS) involvement, as observed in the current study. The increased production of 4-hydroxynonenal via the activation of the AROS axis has been reported in high-fructose diet studies [136]. The soluble RAGE (sRAGE)/RAGE axis has also been shown to be disturbed, and intestinal RAGE expression is shown to be increased (duodenum and colon) in dogs with CIE [137,138,139]. In SYN-treated dogs, multiple genes from the AGE-RAGE signaling pathways exhibited upregulation, with *COL1A1* being notable for its cell-protective effects. For example, the inhibition of *COL1A1* elevates ROS levels within cells, reduces MITO membrane potential, enhances intracellular autophagy, and activates apoptosis [140]. Additionally, the mutation in *Col4a1* leads to ER stress [141].

There is evidence indicating the protective function of *TNF*, *IL5*, and *IL6*, but the subject remains contentious. While the signaling pathway involving TNF and TNF receptor 1 (TNFR1) is associated with promoting inflammatory disease, the TNF receptor 2 (TNFR2) signaling pathway seems to exhibit protective anti-inflammatory effects [142,143]. Research involving murine cardiac myocytes indicates that these cells can leverage TNF–TNFR2 signaling to mitigate ROS production induced by TNF–TNFR1 and avert cell death [144]. Signaling through memTNF, primarily via TNFR2, mainly serves a neuroprotective role by activating NF-κB and AKT-dependent signaling pathways in neurons [145], thereby promoting tissue homeostasis and regeneration. In vitro studies have demonstrated that the neuroprotective properties of TNFR2 necessitate the activation of the PI3K/Akt pathway [145,146]. Dopaminergic neurons exhibit a notable susceptibility to OS due to the involvement of dopamine metabolism and transport in ROS generation. However, these neurons demonstrated resilience against cell death induced by hydrogen peroxide (H_2_O_2_) or 6-hydroxydopamine (6-OHDA) through the selective activation of TNFR2 in response to the toxic challenge [146]. While the precise molecular mechanisms underlying the neuroprotective effects mediated by TNFR2-PI3K/Akt remain unclear, the involvement of Akt and its downstream targets is well established in enhancing cell survival. This is achieved by disrupting cell death pathways, either by inactivating the components of the apoptotic machinery or by activating antiapoptotic proteins [145,147]. Akt can directly inhibit cell death following mitochondrial cytochrome C release, likely through the phosphorylation of caspase 9 at serine 196 [148], which results in the inactivation of the caspase. At a mechanistic level, the activation of TNFR2 facilitates the release of anti-inflammatory and neurotrophic factors [145,149], potentially elucidating some of the protective and regenerative effects associated with TNFR2. Research also indicates that TNFR2 offers protection to oligodendrocyte progenitor cells (OPCs) from OS [150]. The activation of TNFR2 in OPCs leads to an increase in the expression of antiapoptotic and antioxidative proteins, including B-cell lymphoma 2 (BCL-2) and superoxide dismutase 2 (SOD2), which could help maintain the stability of the mitochondrial membrane [151,152]. This mechanism could play a role in the protective effects mediated by TNFR2, particularly in safeguarding OPCs from cell death induced by H_2_O_2_ [150]. While TNF is generated in reaction to infections or oxidative damage, the induced TNF after SYN treatment could stimulate the production of “protective” proteins like MITO manganese superoxide dismutase (MnSOD) [153] and may play a role in alleviating MITO stress following SYN treatment. The possibility of a protective effect due to elevated IL-5 expression resulting from SYN treatment cannot be dismissed. A study indicated a beneficial role of IL-5 in amyotrophic lateral sclerosis (ALS), revealing that individuals with increased IL-5 levels tend to have extended survival [154]. IL-5 shares receptor components with IL-3 and granulocyte–macrophage colony-stimulating factor (GM-CSF), and it is involved in preventing eosinophilic apoptosis during allergen-induced airway inflammation [155]. In Alzheimer’s disease, IL-5 exhibits a protective role by decreasing tau protein hyperphosphorylation and inhibiting cell apoptosis. The activation of the JAK2 pathway plays a vital role in the neuroprotective effects of IL-5 in the context of neurodegeneration [156]. However, further research is required to elucidate the mechanism by which IL-5 influences IBD and its impact on SYN treatment [154]. IL-6, which links inflammatory bowel disease (IBD), AGE-RAGE, JAK/STAT, PI3K/Akt, cytokine–cytokine receptor interaction, and EGFR-TKI-resistance signaling pathways, was upregulated by the SYN treatment versus the PL treatment. The increased expression of IL6 diminishes ROS and contributes to the reduction in oxidative phosphorylation signaling pathways. The reduction in ROS by IL6 correlates with an elevation in the principal antioxidant factor, Nrf2, which swiftly translocates to the MITO to diminish MITO function and promote mitophagy [157]. Moreover, IL-6 has been recognized for its capacity to diminish OS and prevent MITO dysfunction [158]. IL-6 also regulates resistance to radiation by inhibiting OS through the Nrf2-antioxidant pathway [159]. Nrf2, in turn, promotes the expression of *IL-6* through an antioxidant response element found in the *IL-6* promoter [160], suggesting mutual regulation among these signaling pathways. IL-6 also induces a transient but substantial reduction in cellular cAMP levels, possibly facilitating the induction of mitophagy to alleviate ROS effects [157]. Cytokine IL-6 induces the rapid upregulation of signaling pathways in pancreatic β-cells, activating autophagy and antioxidant responses to diminish ROS-mediated cell death, which promotes survival in diabetogenic conditions [157]. The activation of RAGE triggers the production of ROS through NADPH oxidase activation, while additional amplification processes in MITO enhance ROS production [161], causing ER stress and inflammation [162]. ER stress serves as an additional contributor to the elevation of ROS production, potentially worsening OS and MITO impairment, which can result in low-grade inflammation [90]. In contrast, IL18, associated with the inflammatory bowel disease (IBD) signaling pathway, was reduced in SYN-treated CIE dogs. IL18 synergizes with IL-2 to boost cytotoxicity, increase interferon-gamma (IFN-γ) production, and promote the expansion of natural killer cells. IL18 enhances the cytotoxicity of natural killer (NK) and T cells while also boosting the production of other pro-inflammatory cytokines like TNF-alpha (TNF-α), IL-1β, IL-8, and nitric oxide (NO) [163].

The decrease in *GSTT2* levels in CIE dogs following SYN treatment may be attributed to a lowered OS level resulting from the SYN intervention. GSTT2 has been previously demonstrated to safeguard cells from DNA damage after oxidant exposure [164]. The increase in GST was noted as a response to counteracting the oxidants produced in hyperthyroid conditions. The GST levels returned to the baseline as a result of decreased OS in hyperthyroidism when hyperthyroid subjects were administered antioxidants like vitamin E and/or curcumin, as documented in murine studies [97]. A reduction in the quantity of MHC II molecules in antigen-containing compartments following stress has been documented [165], and the upregulation of *DLA-DOA* (*HLA-DOA*) and *DLA-DOB* (*HLA-DOB*) could be a result of reduced stress following SYN treatment. The increase in CREB5 levels may result from the response induced by SYN treatment. The widespread transcription factor, cAMP response element-binding protein (CREB), plays a crucial role in activating the expression of nuclear CRE-regulated genes, which have been demonstrated to be involved in various cellular processes, such as apoptosis and OS. The enhancement of gene expression in both nuclear and MITO proteins associated with the oxidative phosphorylation system, influenced by CREB, may serve as a crucial regulatory mechanism for this essential cellular function [166]. Chemokine (C–C motif) ligands, *CCL1* and *CCL19*, exhibited an increase in expression following SYN treatment, whereas *CCL26* showed a decrease. The elevated expression of *CCL1* and *CCL19* may provide protective benefits through antioxidant and anti-inflammatory pathways; however, additional investigation is necessary. Numerous CCLs demonstrate beneficial effects. Research on *CCL2* has indicated its involvement, either directly or via induced metabolic changes, in the regulation of MITO biogenesis and autophagy. The overexpression of *Ccl2* led to a reduction in AMPK activity, and these alterations were linked to diminished oxidative phosphorylation [167]. Furthermore, the prolonged high expression of *Ccl2* enhances the expression of anti-inflammatory genes in metabolic tissues and may diminish the inflammatory response, as evidenced by studies in murine models [167]. It is crucial to recognize that autophagy plays a significant role in the removal of damaged mitochondria, exhibiting robust anti-inflammatory properties and enhancing energy homeostasis.

Several other genes, including *COL6A6*, *EDAR*, *FoxP3*, *FST*, *GREM1*, *MEF2B*, and *NRG1*, which exhibit protective effects, were found to be upregulated following SYN treatment. While collagen VI demonstrates a wide array of cytoprotective effects, including the ability to mitigate apoptosis and oxidative injury [168], the ectodysplasin-A2 receptor (EDA2R), belonging to the TNF receptor family, exhibits anti-inflammatory and antioxidant properties [169]. Whereas FoxP3 functions as a tumor suppressor with FoxP3+ Tregs, exhibiting protective effects [170], and has been demonstrated to eliminate macrophages and monocytes while inhibiting their protumor activities [171], follistatin (FST) serves to inhibit the production of ROS with *FST* transcription initiation facilitated by Nrf2 [172]. Similarly, *GREM1* demonstrated a protective effect against cell death, as indicated by enhanced survival rates and reduced cytotoxicity in hMPCs overexpressing *GREM1*. Moreover, the overexpression of *GREM1* resulted in the induction of cytoprotective properties through the reduction in ROS and the MITO membrane potential. This outcome was linked to the heightened expression of antioxidant enzymes, as well as the activation of the ERK/NRF2 survival signaling pathway [173]. Likewise, pro-survival transcription factor MEF2 activity plays a crucial role in promoting survival and preventing apoptosis in cardiac myocytes [174] and in cultured primary neurons [175]. Similarly, NRG-1 can diminish ROS production by inhibiting NOX4 via extracellular-regulated protein kinase (ERK) 1/2 and also suppresses the NLR family pyrin domain containing 3 (NLRP3)/caspase-1 pathway, thereby reducing inflammation and oxidative injury [176].

Several FGF subfamily members, such as *FGF2*, *FGF7*, and *FGF10*, which link the Ras, PI3K/Akt, and Rap1 signaling pathways, and *FGF2*, which connects to the EGFR-TKI resistance pathway, were upregulated following SYN treatment. The increased expression of FGFs associated with the Ras signaling pathway contributes to the reduction in OS, which preserves MITO and ER integrity. The Ras/MAPK pathway exhibits spatial compartmentalization within cells, with its signal transduction occurring at the level of the cytoplasm and other intracellular membranes such as the ER, endosomes, and the Golgi apparatus [177]. Ras has shown the ability to protect cells from apoptosis either by activating PKB/Akt via PI3-kinase or by stimulating *NF-κB* [178]. Ras proteins in eukaryotes serve as a key convergence point for various signaling pathways. The plasma membrane proteins Ras1 and Ras2 are responsible for sensing the nutritional conditions of the environment, as reported in *Saccharomyces cerevisiae* [179]. The activation of Ras, Myc, and p53 induces MITO dysfunction, leading to the formation of MITO ROS and downstream signaling (e.g., NF-kB, STAT3), which promote inflammation [180]. The cAMP-protein kinase A pathway is regulated by Ras proteins and influences numerous genes that play a pivotal role in protecting the cell from OS. FGF2, FGF7, and FGF10 have been recognized as essential proteins for providing protective anti-oxidative effects. In vitro studies in murine mesenchymal stem cells (MSCs) demonstrate that FGF2 protects against OS through the regulation of a twist2-p53 signaling axis [181]. Specifically, FGF2 inhibits MITO ROS levels, as well as the expression of p53 and BAX proteins [181]. FGF7 supports redox homeostasis by facilitating the localization of MITO hexokinase2 (HXK2) and the nuclear translocation of Nrf2. This overexpression of FGF7 activates Nrf2 and enhances the scavenging of ROS, ameliorating OS, primarily regulated by PI3Kα/Akt signaling pathway [182]. Another member of the FGF7 subfamily, FGF10, influences several essential cellular processes, including apoptosis, ER stress, and inflammation. The activation of the PI3K/Akt pathway has been identified as a crucial mechanism through which FGF10 exerts protection in tissues by reducing OS and apoptosis [183]. At the cellular level, FGF10 reduces ROS production, apoptosis, DNA damage, and MITO dysfunction via the activation of the Nrf2 pathway and the inhibition of the NF-κB pathway [184]. 

Following SYN treatment, there was an upregulation of *RASGRP2*, Ras association domain family member 5 (*RASSF5*), and *CSF1*, which are associated with the Ras and Rap1 signaling pathways. Additionally, *CSF1* and *CSF3* (linked to the PI3K/Akt pathway), cytokine–cytokine receptor interactions, and *CSF3* associated with the JAK/STAT signaling pathway were also upregulated. We also observed an upregulation of *SynGAP1*, *RASGRP2*, and *RASGRP3* genes, aligning with findings from previous studies that highlight their protective effects. Wnt-associated SynGAP1 has been identified as a neuroprotective agent for glutamatergic synapses and provides defense against the detrimental impacts of Aβ oligomers [185]. RASGRP2 can activate Rap1, which inhibits ROS generated through NOX (NADPH oxidase) in response to TNF-α stimulation and reduces apoptosis [186]. RasGRP3 plays a crucial role in modulating the response of Toll-like receptors (TLRs) by inhibiting the production of pro-inflammatory cytokines using macrophages through the activation of Rap1 small GTPase [187]. p21-activated kinase (PAK)1 to 3 are a group of highly homologous Ser/Thr protein kinases that function as effectors for Rac and Cdc42 [188]. PAK plays a critical role in regulating NF-κB activation by reducing the sensitivity of cells to ROS [189]. Increased levels of THBS1 (from TGF-beta and Rap1 signaling pathways) were also observed in response to SYN treatment. THBS1 functions within ER to activate PKR-like ER kinase (PERK) and Nrf2, which induce a protective antioxidant defense response against the fatty acid palmitate [190]. Consequently, THBS1 has been identified as a protective regulator of ER stress and antioxidant responses in pancreatic β-cells [190]. *RASSF5* exhibited increased expression in CIE dogs post-SYN treatment and might provide protective effects to the colon mucosa. *RASSF1A* plays an important role in regulating the Ras signaling of cellular ROS levels that cause DNA damage [191]. Rassf1a-knockout mice exhibit clinicopathological abnormalities of IBD (increases in intestinal permeability, production of cytokines and chemokines, NF-κB overexpression, and epithelial cell damage), affirming the protective function of RASSFs [192]. Transforming growth factor-beta (TGF-β) regulates OS through increased ROS generation and the modulation of the antioxidative system. However, TGF-β is a pleiotropic cytokine that plays a role in both inhibitory and inflammatory immune responses. Tissue ROS also influences Smad signaling to increase the resistance of cancer cells against TGF-β-mediated proliferation inhibition [193]. ROS activates the mitogen-activated protein kinase (MAPK) pathways to enhance the transcriptional activity of NF-κB, indicating interactions between ROS and TGF-β receptors [194].

SYN-treated dogs also showed an upregulation of HGF, which links Ras, PI3K/Akt, and Rap1 signaling pathways and EGFR-TKI resistance. There are also interactions between HGF and the chemical carcinogenesis–ROS, IL21R, and JAK/STAT signaling pathways. HGF can modulate redox homeostasis through various mechanisms: by influencing the secretion of cytotoxic growth factors such as TGF-β and by inhibiting the activity and expression of NADPH oxidase [195]. Also, HGF drives antioxidant responses by enhancing catalase and SOD1 activities through the activation of NF-κB and the induction of GSH systems [195]. An increased GSH/GSSG (redox) ratio corresponds to the enhanced expression of key enzymes associated with GSH, such as γ-GCS, GPx, GSH-S-transferase, and glucose-6-phosphate dehydrogenase [40,63,195]. HGF and its receptor c-Met exhibit significant antioxidant properties and repair mechanisms in epithelial tissues. This process is linked to the activation of NF-κB and Nrf2, which promotes the expression of antioxidant enzymes and increased glutathione synthesis [196]. The main process of HGF repair is facilitated by canonical signaling pathways, including Akt, STAT3, and extracellular signal-regulated kinase 1/2 (Erk1/2), that depend on cellular redox regulation [196,197]. Moreover, HGF enhances an anti-inflammatory response, as shown by the increase in anti-inflammatory macrophages (CD163^+^) following HGF treatment in murine models [197]. Interleukin-21 (IL21) transmits signals via a receptor complex that includes the IL-21R and the common cytokine receptor γ chain (γc, CD132) [198]. The dimerization of IL-21R and γc results in the recruitment and phosphorylation of JAK1 and JAK3, which then phosphorylate and activate STAT3, STAT1, and, to a lesser extent, STAT5 [198]. Importantly, IL-21 demonstrates beneficial impacts on T-cell MITO health by decreasing ROS production and increasing the production of antioxidants (e.g., glutathione synthesis and catalase) [199]. Moreover, the presence of interleukin-21 (IL21) also enhances fatty acid synthesis and MITO biogenesis, as well as antioxidant formation [199].

Platelet-derived growth factor receptor beta (*PDGFR-β*) and 5-hydroxytryptamine receptor 7 (*HTR7*), linked to the Ras signaling pathway, were also upregulated following SYN treatment. The 5-HTR7 (5-hydroxytryptamine receptor 7) receptors have anti-inflammatory properties, which include a reduction in cell death, OS, and the release of pro-inflammatory cytokines, and they alter the expression of cyclooxygenase (COX) mRNA [200]. The administration of the 5-HT7 receptor agonist LP44 resulted in a reduction in malondialdehyde (MDA) and TNF-α levels and the expression of caspase 3 and caspase 9 mRNA while enhancing SOD activities and GSH levels [201]. 5-HTR7 plays a role in the activation of ERK and Akt, which protect against oxidative injury [202]. Similarly, PDGFR-β is effective in activating the PI3-K/Akt, which mediates the neuroprotective effects of platelet-derived growth factor (PDGF)-B. While both PDGF-A and -B activate the PI3-K and MAPK pathways, PDGF-B exhibits greater neuroprotection by decreasing apoptosis [203]. RAS p21 protein activator 3 (RASA3) functions as a Ras GTPase-activating protein, which is pivotal in regulating ROS levels during terminal erythroid differentiation and preventing apoptosis in precursor cells [204]. EGFR promotes various cellular processes, including the survival, growth, and differentiation of cells. The relationship between ROS and EGFRs in tumor progression and drug resistance has been documented [205]. The overactivation of NADPH oxidase, ROS-induced OS resulting from MITO dysfunction, and ectopic expression of antioxidative enzymes play a role in EGFR-mediated tumor progression and drug resistance to EGFR TKIs [205]. The oxidation of both EGFR and downstream phosphatases by ROS enhances EGFR-mediated signaling and contributes to tumor progression. Various mechanisms of resistance to EGFR-TKIs include alterations in downstream pathways (such as Akt mutations), and disruptions in apoptosis induced by EGFR-TKIs may contribute to the protective effects of SYN treatment in dogs with CIE. Erlotinib, an EGFR-TKI, also has the potential to induce OS in cancer cells by upregulating NOX-4 [206].

The JAK/STAT system is a well-established signaling pathway that modulates mucosal immunological tolerance, contributing to the pathogenesis of IBD [207]. The JAK/STAT pathway plays a pivotal role in regulating the balance between effector and regulatory T cell populations, contributing to robust intestinal immunity. In human IBD, pro-inflammatory cytokines transmit their signals via cytoplasmic JAKs, which, upon phosphorylation, interact with the cytoplasmic proteins known as STATs. STATs then undergo phosphorylation and move into the nucleus, where they promote the transcription of specific target genes, such as *TGF-β*, *TNF-α*, *IL-6*, intercellular adhesion molecule 1 (*ICAM-1*), *STAT1*, and *STAT3* [208]. Dogs with CIE exhibit increased JAK/STAT signaling in inflamed duodenal tissues [209]. However, in the present study, several genes (*IL-6*, *CSF1*, *CSF3*, and *IL-21R*) known for their protective roles in JAK/STAT signaling were upregulated in dogs treated with SYN. Also, oncostatin M (*OSM*), a pleiotropic cytokine and an IL-6 family member, was upregulated with SYN treatment, which can activate inflammatory pathways, including JAK/STAT and PI3K/Akt [210]. Furthermore, while OSM was increased in expression, other genes (*IL-6*, *CSF1*, *CSF3*, and *IL-21R*) were also upregulated, offering protection against inflammation with SYN treatment. *CSF1* has a protective role against cell injury caused by oxidants and reduces OS levels. Osteocyte apoptosis in *CSF-1*KO mice correlates with increased levels of Nox4 and 4-HNE expression in osteocytes. Additionally, CSF1 reduces Nox4 levels in cultured normal osteoblasts, indicating that CSF1 may provide a protective effect against OS [211]. Upregulated CSF3 has comparable beneficial effects to the granulocyte colony-stimulating factor (G-CSF) in reducing OS-induced apoptosis in vascular endothelial cells seen with oxygen-induced retinopathy [212].

Rap1 signaling plays an important role in inhibiting Ras-generated ROS, safeguarding T lymphocytes from OS. Studies show that there is a reduction in nitric oxide (NO) bioavailability and increased ROS generation in Rap1-deficient endothelial cells [213]. Activated Rap1 functions as a negative regulator of cardiac MITO ROS generation [214]. Furthermore, in choroid epithelial cells, Rap1 has the capability to inhibit NOX-dependent ROS activation [215]. Several genes in the Rap1 signaling pathway, including *FGF2*, *FGF10*, and *RASGRP2*, were upregulated, suggesting beneficial effects in response to SYN treatment. RASGRP2 plays a role in inhibiting apoptosis through the reduction in ROS generation, as observed in vascular endothelial cells [186]. RASGRP2 activates Rap1, which inhibits ROS production via NOX-TNF-α stimulation and also reduces apoptosis [186]. TNF-α causes pro-inflammatory changes through ROS generation caused by TNF-α-induced NADPH oxidase activation, which has been shown to activate NF-κB [216]. The PI3K/Akt/mTOR (PAM) signaling pathway represents a highly conserved signal transduction network in eukaryotic cells that facilitates cell survival, growth, and cell cycle progression [217]. Lysophosphatidic acid receptor 3 (LPAR3) involvement in the PI3K/Akt and the Rap1 signaling pathways enhances MITO homeostasis in response to OS [218]. Lysophosphatidic acid (LPA), a growth factor-like lipid mediator, mitigates OS through its type 3 receptor, LPAR3, which plays a vital role in regulating calcium transport from the ER to the mitochondria [218]. The reduction in LPAR3 increases the cytochrome c release induced by cisplatin, suggesting that LPAR3 plays a critical role in inhibiting the MITO apoptosis pathway [218]. In the current investigation, the increase in LPAR3 was not observed following the SYN treatment of CIE dogs. However, *COL6A6* (from PI3K/Akt signaling pathway), which has protective effects, was found to be upregulated. It is important to recognize that NF-κB exhibits both antioxidant and prooxidant functions when responding to OS [219]. The normal activation of *NF-κB* and the associated regulation of autophagy can lead to beneficial outcomes, including reduced ROS accumulation. The relationship between NF-κB and Nrf-2 transcription factors is closely linked, varying according to the specific cell types in which OS occurs. The lack of Nrf-2 typically leads to enhanced NF-κB activity, which plays a role in exacerbating inflammation [86]. Similarly, *NF-κB* can also influence the transcriptional activity of *Nrf-2* [220].

Various hydrolyzed diets have been formulated to manage Crohn’s disease in humans [221,222] and CIE in dogs [223]. These diets are regarded as hypoallergenic due to the hydrolysis process, which alters protein structures to reduce existing allergens and allergenic epitopes, thus making the diets less likely to stimulate an immune response [224,225]. Hydrolyzed diets demonstrate significant clinical efficacy for the prolonged management of both food-responsive diarrhea (FRD) and IBD [37]. Also, the serum metabolome of dogs exhibited notable changes following the introduction of a hydrolyzed diet. However, treatment with prebiotics and glycosaminoglycans in combination with the hydrolyzed diet resulted in a greater increase in lipid metabolites [226]. In the present study, while both treatment groups exhibited similar ultrastructural pathology at diagnosis, only the SYN-treated dogs demonstrated a significant reduction in ER and MITO ultrastructure injury scores and improved MVL compared to their pre-treatment values, as well as in comparison to the PL group (with hydrolyzed protein diet only).

All observed changes suggest that dietary SYN is significant in regulating OS and immune function in the colon. In conclusion, this study highlights the value of employing EM as a useful adjunct to light microscopy, potentially aiding in therapeutic decision-making and evaluating deep mucosal healing. Moreover, the current research also emphasizes the beneficial properties of SYN-containing probiotic strains, prebiotics, and specific IgY, which may arise from the upregulation of protective genes such as *FGF2*, *FGF7*, *FGF10*, *SynGAP1*, *RASGRP2*, *RASGRP3*, *THBS1*, *CSF1*, *CSF3*, *IL21R*, *COL6A6*, *EDAR*, *FoxP3*, *FST*, *GREM1*, *MEF2B*, *NRG1*, *COL1A1*, *HGF*, *HTR7*, and *PDGFR-β*, alongside the downregulation of genes linked to negative effects, including *PRKACB*, *PLA2G12B*, *CALM1*, and *CALM2*, thus demonstrating the protective effects of SYN in comparison to PL regarding maintaining MITO and ER ultrastructural integrity or the reduction in OS, as marked by reduced MDA levels. However, because various other coding and non-coding genes, along with miRNAs, have been shown to be modulated by epigenetic mechanisms like DNA methylation, histone, and RNA modifications, further exploration into the relationships between SYN, diet, gut microbiota, and the epigenome is essential to reveal the mechanisms and potential novel treatment avenues for CIE.

## Figures and Tables

**Table 1:** Qualitative criteria for the assessment of ER ultrastructural changes in dogs with CIE.

Score	Morphologic Appearance
1	Normal
2	Some normal ER with several distended ER
3	Few normal ER with many largely distended ER
4	Majority of ER are distended or blown out

CIE = Chronic inflammatory enteropathy; ER = endoplasmic reticulum.

**Table 2:** Qualitative criteria for assessment of MITO ultrastructural changes in dogs with CIE.

Score	Morphologic Appearance
1	Normal cristae, size, shape
2	Some distended cristae with slight architectural voids
3	Distended cristae, larger voids with some cristae visible
4	Major distended cristae, large voids with few visible cristae

CIE = Chronic inflammatory enteropathy; MITO = mitochondria.

**Table 3:** Genes in common across different signaling pathways.

Pathways	Gene ID	Gene Name	Gene Description	log2 (Fold Change)	*p*
Ras signaling pathway, Rap1 signaling pathway	ENSCAFG00000006861	*ANGPT4*	Angiopoietin-4	1.458	0.045
	ENSCAFG00000005970	*FGFR1*	Fibroblast growth factor receptor-1	1.121	0.033
	ENSCAFG00000006701	*FLT1*	Fms-related receptor tyrosine kinase-1	1.002	0.02
	ENSCAFG00000013148	*NMDE2* (*GRIN2B*)	Glutamate ionotropic receptor NMDA type subunit 2B	6.064	0.042
	ENSCAFG00000014309	*RASGRP2*	RAS guanyl-releasing protein-2	1.939	0.035
	ENSCAFG00000005870	*RASGRP3*	RAS guanyl-releasing protein-3	1.47	0.018
	ENSCAFG00000029799	*RASSF5*	Ras association domain family member-5	1.585	0.02
Inflammatory bowel disease pathway, cytokine–cytokine receptor interaction	ENSCAFG00000028874	*C6L8D7* (*IL-17A*)	Interleukin-17A	5.77	0.018
	ENSCAFG00000002183	*IL-17F*	Interleukin-17F	3.278	0.022
Oxidative phosphorylation, ROS–chemical carcinogenesis	ENSCAFG00000019536	*ATP5D* (*ATP5F1D*)	ATP synthase F1 subunit delta	−0.834	0.04
	ENSCAFG00000016880	*ATP5G1* (*ATP5MC1*)	ATP-synthase membrane subunit c locus 1	−0.981	0.039
	ENSCAFG00000022729	*ATP6* (*MT-ATP6*)	Mitochondrially encoded ATP synthase membrane subunit 6	−1.237	0.029
	ENSCAFG00000002387	*B7ZDP5* (*COX5B*)	Cytochrome c oxidase subunit 5B	−1.121	0.016
	ENSCAFG00000029155	*COX7A2*	Cytochrome c oxidase subunit 7A2	−1.058	0.009
	ENSCAFG00000014738	*COX8A*	Cytochrome c oxidase subunit VIIIA (ubiquitous)	−0.902	0.047
	ENSCAFG00000004842	*NDUFC2*	NADH:ubiquinone oxidoreductase subunit C2	−0.83	0.033
	ENSCAFG00000006913	*LOC612644*	Cytochrome c oxidase subunit 6B1	−1.08	0.015
	ENSCAFG00000009698	*NDUFB8*	NADH:ubiquinone oxidoreductase subunit B8	−0.897	0.024
	ENSCAFG00000017667	*NDUFAB1*	NADH:ubiquinone oxidoreductase subunit AB1	−0.918	0.022
	ENSCAFG00000025112	*NDUFA5*	NADH:ubiquinone oxidoreductase subunit A5	−1.201	0.004
	ENSCAFG00000031458	*LOC477508*	Cytochrome c oxidase subunit 6A1, mitochondrial	−0.963	0.0301
	ENSCAFG00000005861	*NDUFA2*	NADH:ubiquinone oxidoreductase subunit A2	−0.957	0.016
	ENSCAFG00000000982	*NDUFA6*	NADH:ubiquinone oxidoreductase subunit A6	−0.808	0.039
	ENSCAFG00000018497	*NDUFA7*	NADH dehydrogenase [ubiquinone]-1 alpha subcomplex subunit 7	−0.913	0.027
	ENSCAFG00000030171	*NDUFA8*	NADH:ubiquinone oxidoreductase subunit A8	−0.912	0.042
	ENSCAFG00000018753	*NDUFA11*	NADH:ubiquinone oxidoreductase subunit A11	−1.13	0.005
	ENSCAFG00000006232	*NDUFA12*	NADH:ubiquinone oxidoreductase subunit A12	−0.909	0.028
	ENSCAFG00000003930	*NDUFB2*	NADH:ubiquinone oxidoreductase subunit B2	−0.849	0.028
	ENSCAFG00000058755	*NDUFB3*	NADH dehydrogenase [ubiquinone] 1 beta subcomplex subunit 3	−0.979	0.021
	ENSCAFG00000011204	*NDUFB4*	NADH:ubiquinone oxidoreductase subunit B4	−0.793	0.045
	ENSCAFG00000004456	*NDUFB6*	NADH:ubiquinone oxidoreductase subunit B6	−0.93	0.017
	ENSCAFG00000052422	*NDUFS4*	NADH:ubiquinone oxidoreductase subunit S4	−1.099	0.01
	ENSCAFG00000030152	*NDUFS6*	NADH:ubiquinone oxidoreductase subunit S6	−0.939	0.03
	ENSCAFG00000019529	*NDUFS7*	NADH:ubiquinone oxidoreductase core subunit S7	−0.956	0.029
	ENSCAFG00000011083	*NDUFS8*	NADH:ubiquinone oxidoreductase core subunit S8	−0.993	0.022
	ENSCAFG00000022732	*NU3M* (*MT-ND3*)	Mitochondrially encoded NADH:ubiquinone oxidoreductase core subunit 3	−1.23	0.004
	ENSCAFG00000015832	*Q0QEY4* (*SDHB*)	Succinate dehydrogenase complex iron sulfur subunit B	−1.01	0.017
	ENSCAFG00000004260	*UQCRH*	Cytochrome b-c1 complex subunit 6	−0.98	0.019
	ENSCAFG00000000919	*UQCRQ*	Ubiquinol-cytochrome c reductase complex III subunit VII	−0.952	0.023
NF-κB signaling pathway, cytokine–cytokine receptor interaction	ENSCAFG00000000515	*TNFB* (*LTA*)	Lymphotoxin alpha	6.939	0.007
	ENSCAFG00000001005	*TNFRSF13C*	TNF receptor superfamily member 13C	2.822	0.042
	ENSCAFG00000004675	*TNFSF11*	TNF superfamily member 11	1.798	0.017
NF-κB signaling pathway, PI3K/Akt signaling pathway	ENSCAFG00000002171	*B8XNP7* (*SYK*)	Spleen-associated tyrosine kinase	1.634	0.019
Rap1 signaling pathway, cGMP-PKG signaling pathway	ENSCAFG00000016413	*GNAI1*	G-protein subunit alpha i1	1.208	0.013
AGE-RAGE, NF-κB signaling pathway	ENSCAFG00000031460	*ICAM-1*	Intercellular Adhesion Molecule 1	1.828	0.005
TRP channels, cGMP-PKG signaling pathway	ENSCAFG00000023463	*ITPR1*	Inositol-1,4,5-trisphosphate receptor type 1	0.806	0.035
	ENSCAFG00000029786	*Q9BDQ4* (*Bdkrb2*)	B2 bradykinin receptor	1.924	0.0003
PI3K/Akt signaling pathway, Rap1 signaling pathway	ENSCAFG00000004366	*MAGI2*	Membrane-associated guanylate kinase	1.932	0.012
JAK/STAT signaling pathway, PI3K/Akt signaling pathway	ENSCAFG00000015159	*JAK3*	Janus kinase 3	1.842	0.019
PI3K/Akt signaling pathway, cGMP-PKG signaling pathway	ENSCAFG00000003005	*CREB5*	CAMP-responsive element binding protein 5	2.16	0.013
	ENSCAFG00000017387	*PIK3R5*	Phosphoinositide-3-kinase regulatory subunit 5	1.522	0.034
Ras signaling pathway, TRP channels	ENSCAFG00000020319	*PRKACB*	Protein kinase cAMP-activated catalytic subunit beta	−0.81	0.048
AGE-RAGE, PI3K/Akt signaling pathway	ENSCAFG00000014345	*Q28252* (*FN1*)	Fibronectin 1 ED-A	2.038	0.0002
EGFR-TKI resistance, TGF-β signaling pathway	ENSCAFG00000011590	*RPS6KB2*	Ribosomal protein S6 kinase B2	−0.882	0.031
cGMP-PKG signaling pathway, ROS–chemical carcinogenesis	ENSCAFG00000007596	*SLC25A4*	Solute carrier family 25 member 4	−0.944	0.04
AGE-RAGE, NF-κB signaling pathway	ENSCAFG00000020004	*VCAM1*	Vascular cell adhesion molecule 1	3.13	0.0002
TGF-β signaling pathway, Rap1 signaling pathway	ENSCAFG00000008704	*THBS1*	Thrombospondin 1	1.293	0.026
	ENSCAFG00000007045	*ID1*	Inhibitor of DNA binding 1	−0.872	0.049
PI3K/Akt signaling pathway, cGMP-PKG signaling pathway	ENSCAFG00000017382	*PIK3R6*	Phosphoinositide-3-kinase regulatory subunit 6	1.715	0.024
TGF-β signaling pathway, cytokine–cytokine receptor interaction	ENSCAFG00000014880	*BMP4*	Bone morphogenetic protein 4	1.772	0.001
	ENSCAFG00000012011	*BMP7*	Bone morphogenetic protein 7	1.266	0.039
	ENSCAFG00000010107	*BMPR1B*	Bone morphogenetic protein receptor type 1B	6.562	0.0009
NF-κB signaling pathway, cytokine–cytokine receptor interaction	ENSCAFG00000031735	*C4NZX1* (*TNFSF13B*)	Tumor-necrosis factor superfamily member 13b	1.699	0.026
	ENSCAFG00000001970	*EDAR*	Ectodysplasin A receptor	3.037	0.033
	ENSCAFG00000001954	*CCL19*	C-C motif chemokine ligand 19	3.286	0.012
JAK/STAT signaling pathway, cytokine–cytokine receptor interaction	ENSCAFG00000011034	*CRLF2*	Cytokine receptor-like factor 2	1.947	0.038
	ENSCAFG00000001519	*CSF2RB*	Colony-stimulating factor 2 receptor subunit beta	1.486	0.005
	ENSCAFG00000031295	*CSF2RA*	Colony-stimulating factor 2 receptor subunit alpha	1.941	0.011
	ENSCAFG00000018579	*GHR*	Growth-hormone receptor	1.185	0.0131
	ENSCAFG00000018230	*IL13RA2*	Interleukin-13 receptor subunit alpha-2	1.774	0.016
	ENSCAFG00000012844	*IL10RA*	Interleukin-10 receptor subunit alpha	1.83	0.007
	ENSCAFG00000005991	*IL5RA*	Interleukin-5 receptor subunit alpha	2.389	0.046
	ENSCAFG00000018600	*LEPR*	Leptin receptor	1.778	0.004
	ENSCAFG00000018661	*LIFR*	Leukemia inhibitory factor receptor subunit alpha	0.928	0.042
	ENSCAFG00000018648	*OSMR*	Oncostatin M receptor	1.436	0.013
	ENSCAFG00000030931	*Q8SPY1* (*LIF*)	Leukemia inhibitory factor	1.41	0.009
AGE-RAGE signaling pathway, cytokine–cytokine receptor interaction	ENSCAFG00000018349	*CCL2*	C-C motif chemokine ligand 2	2.134	0.006
TRP channels, Rap1 signaling pathway and cGMP-PKG signaling pathway	ENSCAFG00000001097	*ADCY8*	Adenylate cyclase 8	4.351	0.021
Ras signaling pathway, PI3K/Akt signaling pathway, Rap1 signaling pathway	ENSCAFG00000014923	*FGF7*	Fibroblast-growth factor 7	2.357	0.0074
	ENSCAFG00000032695	*FGF10*	Fibroblast-growth factor 10	2.502	0.0001
	ENSCAFG00000002065	*KIT*	Mast/stem cell growth factor receptor Kit	1.602	0.0009
AGE-RAGE, TRP channels, cGMP-PKG signaling pathway	ENSCAFG00000002610	*PRKCE*	Protein kinase C epsilon	1.099	0.005
Ras signaling pathway, Rap1 signaling pathway, EGFR-TKI resistance	ENSCAFG00000002079	*Q2XPT7* (*KDR*)	Vascular endothelial growth factor receptor 2	1.11	0.005
	ENSCAFG00000010881	*Q6QHH2* (*IGF1R*)	Insulin-like growth factor 1 receptor	0.845	0.026
Inflammatory bowel disease, AGE-RAGE, TGF-β signaling pathway	ENSCAFG00000017388	*SMAD3*	SMAD family member 3	0.85	0.039
JAK/STAT signaling pathway, PI3K/Akt signaling pathway, cytokine–cytokine receptor interaction	ENSCAFG00000016261	*CSF3*	Colony-stimulating factor 3	3.963	0.022
	ENSCAFG00000005210	*D2XMM7* (*IL2RA*)	Interleukin-2 receptor subunit alpha	1.722	0.034
Inflammatory bowel disease pathway, AGE-RAGE signaling pathway, cytokine–cytokine receptor interaction	ENSCAFG00000007245	*IL1A*	Interleukin-1 alpha	1.603	0.022
Inflammatory bowel disease pathway, JAK/STAT signaling pathway, cytokine–cytokine receptor interaction	ENSCAFG00000032590	*IL21R*	Interleukin-21 receptor	3.525	0.013
	ENSCAFG00000018542	*IL23R*	Interleukin-23 receptor	2.128	0.022
	ENSCAFG00000000855	*IL5*	Interleukin-5	2.733	0.037
PI3K/Akt signaling pathway, cytokine–cytokine receptor interaction, JAK/STAT signaling pathway	ENSCAFG00000012468	*OSM*	Oncostatin M	7.424	0.0009
AGE-RAGE signaling pathway, TGF-β signaling pathway, cytokine–cytokine receptor interaction	ENSCAFG00000005449	*TGFBR2*	Transforming growth factor beta receptor 2	1.157881378	0.018
NF-κB signaling pathway, TRP channels, cytokine–cytokine receptor interaction	ENSCAFG00000002147	*IL1R1*	Interleukin-1 receptor type 1	1.169	0.021
Ras signaling pathway, TRP channels, Rap1 signaling pathway, cGMP-PKG signaling pathway	ENSCAFG00000002646	*CALM2*	Calmodulin 2	−1.003	0.011
	ENSCAFG00000017516	*CALM1*	Calmodulin 1	−0.893	0.042
Ras signaling pathway, PI3K/Akt signaling pathway, Rap1 signaling pathway, EGFR-TKI resistance	ENSCAFG00000003996	*FGF2*	Fibroblast-growth factor 2	2.45	0.004
EGFR-TKI resistance, JAK/STAT signaling pathway, NF-κB signaling pathway, AGE-RAGE	ENSCAFG00000000068	*BCL2*	BCL2 apoptosis regulator	0.892	0.024
AGE-RAGE, TRP channels, Rap1 signaling pathway, cGMP-PKG signaling pathway	ENSCAFG00000009036	*PLCB2*	Phospholipase C beta 2	1.801	0.0097
Ras signaling pathway, PI3K/Akt signaling pathway, Rap1 signaling pathway, cytokine–cytokine receptor interaction	ENSCAFG00000019798	*CSF1*	Colony-stimulating factor 1	1.555	0.0027
	ENSCAFG00000018219	*CSF1R*	Colony-stimulating factor 1 receptor	1.58	0.034
Inflammatory bowel disease pathway, JAK/STAT signaling pathway, PI3K/Akt signaling pathway, cytokine–cytokine receptor interaction	ENSCAFG00000028978	*IL2RG*	Cytokine receptor common subunit gamma	1.627	0.046
Inflammatory bowel disease pathway, AGE-RAGE signaling pathway, TGF-β signaling pathway, cytokine–cytokine receptor interaction	ENSCAFG00000010815	*Q19KA8* (*TGFbeta2*)	Transforming growth factor beta 2	1.128	0.035
Ras signaling pathway, EGFR-TKI resistance, AGE-RAGE, NF-κB signaling pathway, TRP channels	ENSCAFG00000019989	*PLCG2*	Phospholipase C gamma 2	1.541	0.015
Ras signaling pathway, EGFR-TKI resistance, PI3K/Akt signaling pathway, Rap1 signaling pathway, ROS–chemical carcinogenesis	ENSCAFG00000006370	*HGF*	Hepatocyte growth factor	1.854	0.0025
Ras signaling pathway, EGFR-TKI resistance, JAK/STAT signaling pathway, PI3K/Akt signaling pathway, Rap1 signaling pathway	ENSCAFG00000002057	*PDGFRA*	Platelet-derived growth factor receptor alpha	1.472	0.003
	ENSCAFG00000018214	*PGFRB* (*PDGFRB*)	Platelet-derived growth factor receptor beta	1.753	0.0005
Ras signaling pathway, EGFR-TKI resistance, AGE-RAGE, TRP channels, Rap1 signaling pathway	ENSCAFG00000011326	*PRKCA*	Protein kinase C alpha	0.863	0.046
Ras signaling pathway, EGFR-TKI resistance, AGE-RAGE, TRP channels, Rap1 signaling pathway, NF-κB signaling pathway	ENSCAFG00000009082	*PLCG1*	Phospholipase C gamma 1	0.861	0.046
	ENSCAFG00000017622	*PRKCB*	Protein kinase C beta	1.457	0.038
Inflammatory bowel disease pathway, AGE-RAGE signaling pathway, JAK/STAT signaling pathway, PI3K/Akt signaling pathway, cytokine–cytokine receptor interaction, EGFR-TKI resistance signaling pathway	ENSCAFG00000002733	*Q95LE4* (*IL6*)	Interleukin-6	4.433	0.0028
Ras signaling pathway, EGFR-TKI resistance, AGE-RAGE, JAK/STAT signaling pathway, PI3K/Akt signaling pathway, Rap1 signaling pathway, ROS–chemical carcinogenesis, cGMP-PKG signaling pathway	ENSCAFG00000015806	*Q6PVW1* (*AKT3*)	Protein kinase B gamma-like protein	1.727	0.005

Phosphatidylinositol-3-kinase/protein kinase B (PI3K/Akt). Advanced glycation end products (AGEs). Receptor for advanced glycation end products (RAGEs). Reactive oxygen species (ROS). Janus kinase/signal transducer and activator of transcription (JAK/STAT). Nuclear factor kappa-light-chain-enhancer of activated B cell (NF-κB) signaling pathway. Cyclic guanosine monophosphate (cGMP). Protein kinase G (PKG). Epidermal growth factor receptor (EGFR). Tyrosine kinase inhibitor (TKI). Transforming growth factor beta (TGF-β). Transient receptor potential (TRP).

## Data Availability

All data are available in the tables, figures, and Supplementary Materials.

## Supplementary Materials

The following supporting information can be downloaded at https://www.mdpi.com/article/10.3390/antiox14060727/s1.

## References

[1] Jergens A.E., Heilmann R.M. (2022). Canine Chronic Enteropathy—Current State-of-the-Art and Emerging Concepts. Front. Vet. Sci..

[2] Dandrieux J.R.S., Mansfield C.S. (2019). Chronic Enteropathy In Canines: Prevalence, Impact And Management Strategies. Vet. Med. Res. Rep..

[3] Allenspach K., Wieland B., Gröne A., Gaschen F. (2007). Chronic Enteropathies in Dogs: Evaluation of Risk Factors for Negative Outcome. J. Vet. Intern. Med..

[4] Sahoo D.K., Heilmann R.M., Patel A. (2025). Editorial: Understanding Molecular Mechanisms to Facilitate the Development of Biomarkers for Therapeutic Intervention in Gastrointestinal Diseases and Sepsis. Front. Genet..

[5] Xenoulis P.G., Palculict B., Allenspach K., Steiner J.M., Van House A.M., Suchodolski J.S. (2008). Molecular-Phylogenetic Characterization of Microbial Communities Imbalances in the Small Intestine of Dogs with Inflammatory Bowel Disease. FEMS Microbiol. Ecol..

[6] Honneffer J.B., Minamoto Y., Suchodolski J.S. (2014). Microbiota Alterations in Acute and Chronic Gastrointestinal Inflammation of Cats and Dogs. World J. Gastroenterol..

[7] Rossi G., Pengo G., Caldin M., Piccionello A.P., Steiner J.M., Cohen N.D., Jergens A.E., Suchodolski J.S. (2014). Comparison of Microbiological, Histological, and Immunomodulatory Parameters in Response to Treatment with Either Combination Therapy with Prednisone and Metronidazole or Probiotic VSL#3 Strains in Dogs with Idiopathic Inflammatory Bowel Disease. PLoS ONE.

[8] White R., Atherly T., Guard B., Rossi G., Wang C., Mosher C., Webb C., Hill S., Ackermann M., Sciabarra P. (2017). Randomized, Controlled Trial Evaluating the Effect of Multi-Strain Probiotic on the Mucosal Microbiota in Canine Idiopathic Inflammatory Bowel Disease. Gut Microbes.

[9] Sahoo D.K., Allenspach K., Mochel J.P., Parker V., Rudinsky A.J., Winston J.A., Bourgois-Mochel A., Ackermann M., Heilmann R.M., Köller G. (2022). Synbiotic-IgY Therapy Modulates the Mucosal Microbiome and Inflammatory Indices in Dogs with Chronic Inflammatory Enteropathy: A Randomized, Double-Blind, Placebo-Controlled Study. Vet. Sci..

[10] Day M.J., Bilzer T., Mansell J., Wilcock B., Hall E.J., Jergens A., Minami T., Willard M., Washabau R. (2008). Histopathological Standards for the Diagnosis of Gastrointestinal Inflammation in Endoscopic Biopsy Samples from the Dog and Cat: A Report from the World Small Animal Veterinary Association Gastrointestinal Standardization Group. J. Comp. Pathol..

[11] Willard M.D., Moore G.E., Denton B.D., Day M.J., Mansell J., Bilzer T., Wilcock B., Gualtieri M., Olivero D., Lecoindre P. (2010). Effect of Tissue Processing on Assessment of Endoscopic Intestinal Biopsies in Dogs and Cats. J. Vet. Intern. Med..

[12] Allenspach K.A., Mochel J.P., Du Y., Priestnall S.L., Moore F., Slayter M., Rodrigues A., Ackermann M., Krockenberger M., Mansell J. (2019). Correlating Gastrointestinal Histopathologic Changes to Clinical Disease Activity in Dogs With Idiopathic Inflammatory Bowel Disease. Vet. Pathol..

[13] Laschi R., Pasquinelli G., Versura P. (1987). Scanning Electron Microscopy Application in Clinical Research. Scanning Microsc..

[14] Schattenfroh S., Bartels M., Nagel E. (1994). Early Morphological Changes in Crohn’s DiseaseTransmission Electron-Microscopic Findings and Their Interpretation: An Overview. Acta Anat..

[15] Bertini M., Sbarbati A., Canioni D., Schmitz J. (1998). Scanning Electron Microscopy in Childhood Inflammatory Bowel Disease. Scan. Microsc. Int..

[16] Morroni M., Sbarbati A., D’Angelo G., Catassi C., Giorgi P., Cinti S. (1989). Scanning Electron Microscopy of the Small Intestine Mucosa in Children with Celiac Disease After Long-Term Dietary Treatment. Scanning Microsc..

[17] Aluwihare A.P. (1971). Electron Microscopy in Crohn’s Disease. Gut.

[18] Lewis D., Walker-Smith J.A., Phillips A.D. (1984). Microvilli- and Desmosome-Associated Bodies in Crohn’s Disease and Other Disorders in Childhood: An Ultrastructural Abnormality of the Small and Large Intestine. J. Pediatr. Gastroenterol. Nutr..

[19] Marin M.L., Geller S.A., Greenstein A.J., Marin R.H., Gordon R.E., Aufses A.H. (1983). Ultrastructural Pathology of Crohn’s Disease: Correlated Transmission Electron Microscopy, Scanning Electron Microscopy, and Freeze Fracture Studies. Am. J. Gastroenterol..

[20] Mughal S., Filipe M.I. (1992). Ultrastructural Study of Inflammatory Bowel Disease. Histol. Histopathol..

[21] Myllärniemi H., Nickels J. (1980). Scanning Electron Microscopy of Crohn’s Disease and Ulcerative Colitis of the Colon. Virchows Arch. A Pathol. Anat. Histol..

[22] Nagel E., Bartels M., Pichlmayr R. (1995). Scanning Electron-Microscopic Lesions in Crohn’s Disease: Relevance for the Interpretation of Postoperative Recurrence. Gastroenterology.

[23] Nyhlin H., Stenling R. (1984). The Small-Intestinal Mucosa in Patients with Crohn’s Disease Assessed by Scanning Electron and Light Microscopy. Scand. J. Gastroenterol..

[24] Rickert R.R., Carter H.W. (1980). The “Early” Ulcerative Lesion of Crohn’s Disease : Correlative Light and Scanning Electron-Microscopic Studies. J. Clin. Gastroenterol..

[25] Fratila O.C., Craciun C. (2010). Ultrastructural Evidence of Mucosal Healing after Infliximab in Patients with Ulcerative Colitis. J. Gastrointestin. Liver Dis..

[26] Shields H.M., Bates M.L., Goldman H., Zuckerman G.R., Mills B.A., Best C.J., Bair F.A., Goran D.A., DeSchryver-Kecskemeti K. (1985). Scanning Electron Microscopic Appearance of Chronic Ulcerative Colitis with and without Dysplasia. Gastroenterology.

[27] Nomura E., Sujino T., Hosoe N., Yoshimatsu Y., Tanemoto S., Takabayashi K., Mutaguchi M., Shimoda M., Naganuma M., Ogata H. (2021). Characteristics of the Mucosal Surface on Scanning Electron Microscopy in Patients with Remitting Ulcerative Colitis. Dig. Dis. Sci..

[28] Walker D., Knuchel-Takano A., Mccutchan A., Chang Y.M., Downes C., Miller S., Stevens K., Verheyen K., Phillips A.D., Miah S. (2013). A Comprehensive Pathological Survey of Duodenal Biopsies from Dogs with Diet-Responsive Chronic Enteropathy. J. Vet. Intern. Med..

[29] Shah H., Trivedi M., Gurjar T., Sahoo D.K., Jergens A.E., Yadav V.K., Patel A., Pandya P. (2024). Decoding the Gut Microbiome in Companion Animals: Impacts and Innovations. Microorganisms.

[30] Mounir M., Ibijbijen A., Farih K., Rabetafika H.N., Razafindralambo H.L. (2022). Synbiotics and Their Antioxidant Properties, Mechanisms, and Benefits on Human and Animal Health: A Narrative Review. Biomolecules.

[31] Feng T., Wang J. (2020). Oxidative Stress Tolerance and Antioxidant Capacity of Lactic Acid Bacteria as Probiotic: A Systematic Review. Gut Microbes.

[32] Chaudhary A., Prajapati N., Prajapati A., Singh S., Joshi M., Prajapati D., Patani A., Sahoo D.K., Patel A. (2024). Postbiotic Emissaries: A Comprehensive Review on the Bioprospecting and Production of Bioactive Compounds by Enterococcus Species. Int. J. Food Sci. Technol..

[33] Sahoo D.K., Chainy G.B.N., Jergens A.E. (2025). Editorial: Gastrointestinal (GI) Disorders and Antioxidant Therapeutics. Front. Endocrinol..

[34] Mohammed A.A., Jiang S., Jacobs J.A., Cheng H.W. (2019). Effect of a Synbiotic Supplement on Cecal Microbial Ecology, Antioxidant Status, and Immune Response of Broiler Chickens Reared under Heat Stress. Poult. Sci..

[35] Cukkemane A., Kumar P., Sathyamoorthy B. (2020). A Metabolomics Footprint Approach to Understanding the Benefits of Synbiotics in Functional Foods and Dietary Therapeutics for Health, Communicable and Non-Communicable Diseases. Food Res. Int..

[36] Zheng H.J., Guo J., Jia Q., Huang Y.S., Huang W.J., Zhang W., Zhang F., Liu W.J., Wang Y. (2019). The Effect of Probiotic and Synbiotic Supplementation on Biomarkers of Inflammation and Oxidative Stress in Diabetic Patients: A Systematic Review and Meta-Analysis of Randomized Controlled Trials. Pharmacol. Res..

[37] Allenspach K., Culverwell C., Chan D. (2016). Long-Term Outcome in Dogs with Chronic Enteropathies: 203 Cases. Vet. Rec..

[38] Volkmann M., Steiner J.M., Fosgate G.T., Zentek J., Hartmann S., Kohn B. (2017). Chronic Diarrhea in Dogs – Retrospective Study in 136 Cases. J. Vet. Intern. Med..

[39] Ching T., Huang S., Garmire L.X. (2014). Power Analysis and Sample Size Estimation for RNA-Seq Differential Expression. RNA.

[40] Wong D., Sahoo K.D., Faivre C., Kopper J., Dersh K., Beachler T., Esser M. (2025). Oxidative Stress in Critically Ill Neonatal Foals. J. Vet. Intern. Med..

[41] Canis Lupus Familiaris Genome Assembly Dog10K_Boxer_Tasha-NCBI-NLM. https://www.ncbi.nlm.nih.gov/datasets/genome/GCF_000002285.5/.

[42] Mortazavi A., Williams B.A., McCue K., Schaeffer L., Wold B. (2008). Mapping and Quantifying Mammalian Transcriptomes by RNA-Seq. Nat. Methods.

[43] Liao Y., Smyth G.K., Shi W. (2014). FeatureCounts: An Efficient General Purpose Program for Assigning Sequence Reads to Genomic Features. Bioinformatics.

[44] Krause C., Suwada K., Blomme E.A.G., Kowalkowski K., Liguori M.J., Mahalingaiah P.K., Mittelstadt S., Peterson R., Rendino L., Vo A. (2023). Preclinical Species Gene Expression Database: Development and Meta-Analysis. Front. Genet..

[45] Anders S., Huber W. (2010). Differential Expression Analysis for Sequence Count Data. Genome Biol..

[46] Love M.I., Huber W., Anders S. (2014). Moderated Estimation of Fold Change and Dispersion for RNA-Seq Data with DESeq2. Genome Biol..

[47] Benjamini Y., Hochberg Y. (1995). Controlling the False Discovery Rate: A Practical and Powerful Approach to Multiple Testing. J. R. Stat. Soc. Ser. B (Methodol.).

[48] Young M.D., Wakefield M.J., Smyth G.K., Oshlack A. (2010). Gene Ontology Analysis for RNA-Seq: Accounting for Selection Bias. Genome Biol..

[49] Kanehisa M., Goto S. (2000). KEGG: Kyoto Encyclopedia of Genes and Genomes. Nucleic Acids Res..

[50] Xie C., Mao X., Huang J., Ding Y., Wu J., Dong S., Kong L., Gao G., Li C.Y., Wei L. (2011). KOBAS 2.0: A Web Server for Annotation and Identification of Enriched Pathways and Diseases. Nucleic Acids Res..

[51] Mao X., Cai T., Olyarchuk J.G., Wei L. (2005). Automated Genome Annotation and Pathway Identification Using the KEGG Orthology (KO) as a Controlled Vocabulary. Bioinformatics.

[52] KEGG: Kyoto Encyclopedia of Genes and Genomes. https://www.genome.jp/kegg/.

[53] Chhabria S., Mathur S., Vadakan S., Sahoo D.K., Mishra P., Paital B. (2022). A Review on Phytochemical and Pharmacological Facets of Tropical Ethnomedicinal Plants as Reformed DPP-IV Inhibitors to Regulate Incretin Activity. Front. Endocrinol..

[54] Chattopadhyay S., Sahoo D.K., Roy A., Samanta L., Chainy G.B.N. (2010). Thiol Redox Status Critically Influences Mitochondrial Response to Thyroid Hormone-Induced Hepatic Oxidative Injury: A Temporal Analysis. Cell Biochem. Funct..

[55] Sahoo D.K., Jena S., Chainy G.B.N. (2019). Thyroid Dysfunction and Testicular Redox Status: An Intriguing Association. Oxidants, Antioxidants, and Impact of the Oxidative Status in Male Reproduction.

[56] Patani A., Balram D., Yadav V.K., Lian K.Y., Patel A., Sahoo D.K. (2023). Harnessing the Power of Nutritional Antioxidants against Adrenal Hormone Imbalance-Associated Oxidative Stress. Front. Endocrinol..

[57] Sahoo D.K., Samanta L., Kesari K.K., Mukherjee S. (2024). Editorial: Hormonal Imbalance-Associated Oxidative Stress and Protective Benefits of Nutritional Antioxidants. Front. Endocrinol..

[58] Pati S.G., Panda F., Paital B., Sahoo D.K., Jena S. (2023). Oxidative Stress Physiology in Scylla Serrata for Environmental Health Assessment. Front. Environ. Sci..

[59] Ladumor R., Pandya H., Thakkar M., Mehta D., Paithankar P., Alfarraj S., Ansari M.J., Pandya P., Yadav V.K., Sahoo D.K. (2024). Environmentally Relevant Concentrations of Nickel and Imidacloprid Induce Reproductive Toxicity in Earthworm (Eisenia Fetida Fetida). Comp. Biochem. Physiol. Part. C Toxicol. Pharmacol..

[60] Mishra P., Sahoo D.K., Mohanty C., Samanta L. (2024). Curcumin-Loaded Nanoparticles Effectively Prevent T4-Induced Oxidative Stress in Rat Heart. Cell Biochem. Funct..

[61] Sahoo D.K., Roy A., Chattopadhyay S., Chainy G.B.N. (2007). Effect of T3 Treatment on Glutathione Redox Pool and Its Metabolizing Enzymes in Mitochondrial and Post-Mitochondrial Fractions of Adult Rat Testes. Indian. J. Exp. Biol..

[62] Sahoo D.K., Roy A., Chainy G.B.N. (2008). Rat Testicular Mitochondrial Antioxidant Defence System and Its Modulation by Aging. Acta Biol. Hung..

[63] Sahoo D.K., Chainy G.B.N. (2023). Hormone-Linked Redox Status and Its Modulation by Antioxidants. Vitam. Horm..

[64] Sahoo D.K., Wong D., Patani A., Paital B., Yadav V.K., Patel A., Jergens A.E. (2024). Exploring the Role of Antioxidants in Sepsis-Associated Oxidative Stress: A Comprehensive Review. Front. Cell. Infect. Microbiol..

[65] Sahoo D.K., Heilmann R.M., Paital B., Patel A., Yadav V.K., Wong D., Jergens A.E. (2023). Oxidative Stress, Hormones, and Effects of Natural Antioxidants on Intestinal Inflammation in Inflammatory Bowel Disease. Front. Endocrinol..

[66] Muro P., Zhang L., Li S., Zhao Z., Jin T., Mao F., Mao Z. (2024). The Emerging Role of Oxidative Stress in Inflammatory Bowel Disease. Front. Endocrinol..

[67] Crumeyrolle-Arias M., Jaglin M., Bruneau A., Vancassel S., Cardona A., Daugé V., Naudon L., Rabot S. (2014). Absence of the Gut Microbiota Enhances Anxiety-like Behavior and Neuroendocrine Response to Acute Stress in Rats. Psychoneuroendocrinology.

[68] So S.Y., Savidge T.C. (2022). Gut Feelings: The Microbiota-Gut-Brain Axis on Steroids. Am. J. Physiol. Gastrointest. Liver Physiol..

[69] Sudo N., Chida Y., Aiba Y., Sonoda J., Oyama N., Yu X.N., Kubo C., Koga Y. (2004). Postnatal Microbial Colonization Programs the Hypothalamic-Pituitary-Adrenal System for Stress Response in Mice. J. Physiol..

[70] Vagnerová K., Vodička M., Hermanová P., Ergang P., Šrůtková D., Klusoňová P., Balounová K., Hudcovic T., Pácha J. (2019). Interactions Between Gut Microbiota and Acute Restraint Stress in Peripheral Structures of the Hypothalamic–Pituitary–Adrenal Axis and the Intestine of Male Mice. Front. Immunol..

[71] Gareau M.G., Jury J., MacQueen G., Sherman P.M., Perdue M.H. (2007). Probiotic Treatment of Rat Pups Normalises Corticosterone Release and Ameliorates Colonic Dysfunction Induced by Maternal Separation. Gut.

[72] Lutgendorff F., Akkermans L., Soderholm J. (2008). The Role of Microbiota and Probiotics in Stress-Induced Gastrointestinal Damage. Curr. Mol. Med..

[73] O’Mahony S.M., Marchesi J.R., Scully P., Codling C., Ceolho A.M., Quigley E.M.M., Cryan J.F., Dinan T.G. (2009). Early Life Stress Alters Behavior, Immunity, and Microbiota in Rats: Implications for Irritable Bowel Syndrome and Psychiatric Illnesses. Biol. Psychiatry.

[74] Yang K., Jian S., Wen C., Guo D., Liao P., Wen J., Kuang T., Han S., Liu Q., Deng B. (2022). Gallnut Tannic Acid Exerts Anti-Stress Effects on Stress-Induced Inflammatory Response, Dysbiotic Gut Microbiota, and Alterations of Serum Metabolic Profile in Beagle Dogs. Front. Nutr..

[75] Li Y., Huang B., Sun S., Liu N., Li Y., Lan M., Wang X., Zhang Y., Wu A., Yang S. (2023). Immunoprotection Effects of Chicken Egg Yolk Immunoglobulins (IgY) against Aeromonas Veronii Infection in Sinocyclocheilus Grahami. Aquaculture.

[76] Zhang L., Bai Y., Tao J., Yang S., Tu C., Liu L., Huang X., Li L., Qin Z. (2024). Effects of Feeding Chicken Egg Yolk Antibodies on Intestinal Cell Apoptosis, Oxidative Stress and Microbial Flora of Tilapia (Oreochromis Niloticus) Infected with Streptococcus Agalactiae. Fish. Shellfish. Immunol..

[77] Lu Y., Xiong Y., Zhang S., Wang B., Feng Y., Pu Z., Wei K., Chen J., Chen D., Zhang P. (2024). D-Mannose Reduces Oxidative Stress, Inhibits Inflammation, and Increases Treg Cell Proportions in Mice with Ulcerative Colitis. Front. Pharmacol..

[78] Joshi C.S., Salazar A.M., Wang C., Ligon M.M., Chappidi R.R., Fashemi B.E., Felder P.A., Mora A., Grimm S.L., Coarfa C. (2024). D-Mannose Reduces Cellular Senescence and NLRP3/GasderminD/IL-1β-Driven Pyroptotic Uroepithelial Cell Shedding in the Murine Bladder. Dev. Cell.

[79] Wang J., Motlagh N.J., Wang C., Wojtkiewicz G.R., Schmidt S., Chau C., Narsimhan R., Kullenberg E.G., Zhu C., Linnoila J. (2021). D-Mannose Suppresses Oxidative Response and Blocks Phagocytosis in Experimental Neuroinflammation. Proc. Natl. Acad. Sci. USA.

[80] Dong L., Xie J., Wang Y., Jiang H., Chen K., Li D., Wang J., Liu Y., He J., Zhou J. (2022). Mannose Ameliorates Experimental Colitis by Protecting Intestinal Barrier Integrity. Nat. Commun..

[81] Novak E.A., Mollen K.P. (2015). Mitochondrial Dysfunction in Inflammatory Bowel Disease. Front. Cell Dev. Biol..

[82] Sharma N. (2025). Exploring the Potential of Mannan Oligosaccharides in Enhancing Animal Growth, Immunity, and Overall Health: A Review. Carbohydr. Polym. Technol. Appl..

[83] Lu Z., Feng L., Jiang W., Wu P., Liu Y., Jiang J., Kuang S., Tang L., Li S., Zhong C. (2023). Mannan Oligosaccharides Alleviate Oxidative Injury in the Head Kidney and Spleen in Grass Carp (Ctenopharyngodon Idella) via the Nrf2 Signaling Pathway after Aeromonas Hydrophila Infection. J. Anim. Sci. Biotechnol..

[84] Shaki F., Pourahmad J. (2013). Mitochondrial Toxicity of Depleted Uranium: Protection by Beta-Glucan. Iran. J. Pharm. Res..

[85] Stothers C.L., Burelbach K.R., Owen A.M., Patil N.K., McBride M.A., Bohannon J.K., Luan L., Hernandez A., Patil T.K., Williams D.L. (2021). Beta-Glucan Induces Distinct and Protective Innate Immune Memory in Differentiated Macrophages. J. Immunol..

[86] Kong Y., Olejar K.J., On S.L.W., Chelikani V. (2020). The Potential of Lactobacillus Spp. for Modulating Oxidative Stress in the Gastrointestinal Tract. Antioxidants.

[87] Finamore A., Ambra R., Nobili F., Garaguso I., Raguzzini A., Serafini M. (2018). Redox Role of Lactobacillus Casei Shirota against the Cellular Damage Induced by 2,2’-Azobis (2-Amidinopropane) Dihydrochloride-Induced Oxidative and Inflammatory Stress in Enterocytes-like Epithelial Cells. Front. Immunol..

[88] Serata M., Kiwaki M., Iino T. (2016). Functional Analysis of a Novel Hydrogen Peroxide Resistance Gene in Lactobacillus Casei Strain Shirota. Microbiology.

[89] Yamazaki T., Yamada S., Ohshio K., Sugamata M., Morita Y. (2022). Lactobacillus Paracasei KW3110 Prevents Inflammatory-Stress-Induced Mitochondrial Dysfunction in Mouse Macrophages. Int. J. Mol. Sci..

[90] Tang C., Meng F., Pang X., Chen M., Zhou L., Lu Z., Lu Y. (2020). Protective Effects of Lactobacillus Acidophilus NX2-6 against Oleic Acid-Induced Steatosis, Mitochondrial Dysfunction, Endoplasmic Reticulum Stress and Inflammatory Responses. J. Funct. Foods.

[91] Cuevas-González P.F., Aguilar-Toalá J.E., García H.S., González-Córdova A.F., Vallejo-Cordoba B., Hernández-Mendoza A. (2020). Protective Effect of the Intracellular Content from Potential Probiotic Bacteria against Oxidative Damage Induced by Acrylamide in Human Erythrocytes. Probiotics Antimicrob. Proteins.

[92] Li B., Du P., Smith E.E., Wang S., Jiao Y., Guo L., Huo G., Liu F. (2019). In Vitro and in Vivo Evaluation of an Exopolysaccharide Produced by Lactobacillus Helveticus KLDS1.8701 for the Alleviative Effect on Oxidative Stress. Food Funct..

[93] Petruk G., Donadio G., Lanzilli M., Isticato R., Monti D.M. (2018). Alternative Use of Bacillus Subtilis Spores: Protection against Environmental Oxidative Stress in Human Normal Keratinocytes. Sci. Rep..

[94] Zou X.Y., Zhang M., Tu W.J., Zhang Q., Jin M.L., Fang R.D., Jiang S. (2022). Bacillus Subtilis Inhibits Intestinal Inflammation and Oxidative Stress by Regulating Gut Flora and Related Metabolites in Laying Hens. Animal.

[95] Luo M., Sun J., Li S., Wei L., Sun R., Feng X., Zhang H., Chen T., Xi Q., Zhang Y. (2024). Protective Effect of Enterococcus Faecium against Ethanol-Induced Gastric Injury via Extracellular Vesicles. Microbiol. Spectr..

[96] Merino de Paz N., Carrillo-Palau M., Hernández-Camba A., Abreu-González P., de Vera-González A., González-Delgado A., Martín-González C., González-Gay M., Ferraz-Amaro I. (2024). Association of Serum Malondialdehyde Levels with Lipid Profile and Liver Function in Patients with Inflammatory Bowel Disease. Antioxidants.

[97] Sahoo D.K., Roy A., Chainy G.B.N. (2008). Protective Effects of Vitamin E and Curcumin on L-Thyroxine-Induced Rat Testicular Oxidative Stress. Chem. Biol. Interact..

[98] Candellone A., Girolami F., Badino P., Jarriyawattanachaikul W., Odore R. (2022). Changes in the Oxidative Stress Status of Dogs Affected by Acute Enteropathies. Vet. Sci..

[99] Dvorak A.M., Dickersin G.R. (1979). Crohn’s Disease: Electron Microscopic Studies. Pathol. Annu..

[100] Shiner M., Birbeck M.S.C. (1961). The Microvilli of the Small Intestinal Surface Epithelium in Coeliac Disease and in Idiopathic Steatorrhoea. Gut.

[101] Sbarbati A., Valletta E., Bertini M., Cipolli M., Morroni M., Pinelli L., Tatò L. (2003). Gluten Sensitivity and “normal” Histology: Is the Intestinal Mucosa Really Normal?. Dig. Liver Dis..

[102] Bochimoto H., Kondoh D., Nagata R., Ishihara Y., Tomiyasu J., Han K.H., Shimada K., Sasaki M., Kitamura N., Fukushima M. (2019). Ultrastructural Changes in Colonic Epithelial Cells in a Rat Model of Inflammatory Bowel Disease. Microsc. Res. Tech..

[103] Bou-Fersen A.M., Anim J.T., Khan I. (2008). Experimental Colitis Is Associated with Ultrastructural Changes in Inflamed and Uninflamed Regions of the Gastrointestinal Tract. Med. Princ. Pract..

[104] Manners H.K., Hart C.A., Getty B., Kelly D.F., Sørensen S.H., Batt R.M. (1998). Characterization of Intestinal Morphologic, Biochemical, and Ultrastructural Features in Gluten-Sensitive Irish Setters during Controlled Oral Gluten Challenge Exposure after Weaning. Am. J. Vet. Res..

[105] Kumar V., Bermea K.C., Kumar D., Singh A., Verma A., Kaileh M., Sen R., Lakatta E.G., Adamo L. (2024). RelA-Mediated Signaling Connects Adaptation to Chronic Cardiomyocyte Stress with Myocardial and Systemic Inflammation in the ADCY8 Model of Accelerated Aging. Geroscience.

[106] Han W.Q., Xu L., Tang X.F., Chen W.D., Wu Y.J., Gao P.J. (2018). Membrane Rafts–Redox Signalling Pathway Contributes to Renal Fibrosis via Modulation of the Renal Tubular Epithelial–Mesenchymal Transition. J. Physiol..

[107] Liu Y.S., Hsu J.W., Lin H.Y., Lai S.W., Huang B.R., Tsai C.F., Lu D.Y. (2019). Bradykinin B1 Receptor Contributes to Interleukin-8 Production and Glioblastoma Migration through Interaction of STAT3 and SP-1. Neuropharmacology.

[108] Vilotić A., Nacka-Aleksić M., Pirković A., Bojić-Trbojević Ž., Dekanski D., Jovanović Krivokuća M. (2022). IL-6 and IL-8: An Overview of Their Roles in Healthy and Pathological Pregnancies. Int. J. Mol. Sci..

[109] Chinedu O., Tonassé W.V., Albuquerque D.M., Domingos I.d.F., Araújo A.d.S., Bezerra M.A.C., Sonati M.d.F., dos Santos M.N.N. (2020). Polymorphisms in the Heme Oxygenase-1 and Bone Morphogenetic Protein Receptor Type 1b Genes and Estimated Glomerular Filtration Rate in Brazilian Sickle Cell Anemia Patients. Hematol. Transfus. Cell Ther..

[110] Foret M.K., Orciani C., Welikovitch L.A., Huang C., Cuello A.C., Do Carmo S. (2024). Early Oxidative Stress and DNA Damage in Aβ-Burdened Hippocampal Neurons in an Alzheimer’s-like Transgenic Rat Model. Commun. Biol..

[111] Zhang J., Huang S., Zhu Z., Gatt A., Liu J. (2024). E-Selectin in Vascular Pathophysiology. Front. Immunol..

[112] Lu X., Liu Y., Xuan W., Ye J., Yao H., Huang C., Li J. (2019). Circ_1639 Induces Cells Inflammation Responses by Sponging MiR-122 and Regulating TNFRSF13C Expression in Alcoholic Liver Disease. Toxicol. Lett..

[113] Mushtaq U., Bashir M., Nabi S., Khanday F.A. (2021). Epidermal Growth Factor Receptor and Integrins Meet Redox Signaling through P66shc and Rac1. Cytokine.

[114] Lin S.J., Shyue S.K., Hung Y.Y., Chen Y.H., Ku H.H., Chen J.W., Tam K.B., Chen Y.L. (2005). Superoxide Dismutase Inhibits the Expression of Vascular Cell Adhesion Molecule-1 and Intracellular Cell Adhesion Molecule-1 Induced by Tumor Necrosis Factor-α in Human Endothelial Cells through the JNK/P38 Pathways. Arterioscler. Thromb. Vasc. Biol..

[115] McGeachy M.J., Cua D.J., Gaffen S.L. (2019). The IL-17 Family of Cytokines in Health and Disease. Immunity.

[116] Kleinschek M.A., Boniface K., Sadekova S., Grein J., Murphy E.E., Turner S.P., Raskin L., Desai B., Faubion W.A., De Malefyt R.W. (2009). Circulating and Gut-Resident Human Th17 Cells Express CD161 and Promote Intestinal Inflammation. J. Exp. Med..

[117] Ivanov I.I., Atarashi K., Manel N., Brodie E.L., Shima T., Karaoz U., Wei D., Goldfarb K.C., Santee C.A., Lynch S.V. (2009). Induction of Intestinal Th17 Cells by Segmented Filamentous Bacteria. Cell.

[118] Kumar P., Monin L., Castillo P., Elsegeiny W., Horne W., Eddens T., Vikram A., Good M., Schoenborn A.A., Bibby K. (2016). Intestinal Interleukin-17 Receptor Signaling Mediates Reciprocal Control of the Gut Microbiota and Autoimmune Inflammation. Immunity.

[119] Hueber W., Sands B.E., Lewitzky S., Vandemeulebroecke M., Reinisch W., Higgins P.D.R., Wehkamp J., Feagan B.G., Yao M.D., Karczewski M. (2012). Secukinumab, a Human Anti-IL-17A Monoclonal Antibody, for Moderate to Severe Crohn’s Disease: Unexpected Results of a Randomised, Double-Blindplacebo- Controlled Trial. Gut.

[120] Targan S.R., Feagan B., Vermeire S., Panaccione R., Melmed G.Y., Landers C., Li D., Russell C., Newmark R., Zhang N. (2016). A Randomized, Double-Blind, Placebo-Controlled Phase 2 Study of Brodalumab in Patients with Moderate-to-Severe Crohn’s Disease. Am. J. Gastroenterol..

[121] Lee J.S., Tato C.M., Joyce-Shaikh B., Gulan F., Cayatte C., Chen Y., Blumenschein W.M., Judo M., Ayanoglu G., McClanahan T.K. (2015). IL-23-Independent IL-17 Production Regulates Intestinal Epithelial Permeability. Immunity.

[122] Whibley N., Gaffen S.L. (2015). Gut-Busters: IL-17 Ain’t Afraid of No IL-23. Immunity.

[123] Maxwell J.R., Zhang Y., Brown W.A., Smith C.L., Byrne F.R., Fiorino M., Stevens E., Bigler J., Davis J.A., Rottman J.B. (2015). Differential Roles for Interleukin-23 and Interleukin-17 in Intestinal Immunoregulation. Immunity.

[124] Puel A., Cypowyj S., Bustamante J., Wright J.F., Liu L., Lim H.K., Migaud M., Israel L., Chrabieh M., Audry M. (2011). Chronic Mucocutaneous Candidiasis in Humans with Inborn Errors of Interleukin-17 Immunity. Science (1979).

[125] Hellenthal K.E.M., Brabenec L., Gross E.R., Wagner N.M. (2021). TRP Channels as Sensors of Aldehyde and Oxidative Stress. Biomolecules.

[126] Zheng Z., Li Y., Jin G., Huang T., Zou M., Duan S. (2020). The Biological Role of Arachidonic Acid 12-Lipoxygenase (ALOX12) in Various Human Diseases. Biomed. Pharmacother..

[127] Catanzaro O., Capponi J.A., Michieli J., Labal E., Di Martino I., Sirois P. (2013). Bradykinin B1 Antagonism Inhibits Oxidative Stress and Restores Na+K+ ATPase Activity in Diabetic Rat Peripheral Nervous System. Peptides.

[128] Huang Z., Zhou L., Duan J., Qin S., Jiang J., Chen H., Wang K., Liu R., Yuan M., Tang X. (2024). Oxidative Stress Promotes Liver Cancer Metastasis via RNF25-Mediated E-Cadherin Protein Degradation. Adv. Sci..

[129] Aravind P., Bulbule S.R., Hemalatha N., Babu R.L., Devaraju K.S. (2021). Elevation of Gene Expression of Calcineurin, Calmodulin and Calsyntenin in Oxidative Stress Induced PC12 Cells. Genes. Dis..

[130] Biasi F., Leonarduzzi G., Oteiza P.I., Poli G. (2013). Inflammatory Bowel Disease: Mechanisms, Redox Considerations, and Therapeutic Targets. Antioxid. Redox Signal.

[131] Andresen L., Jørgensen V.L., Perner A., Hansen A., Eugen-Olsen J., Rask-Madsen J. (2005). Activation of Nuclear Factor KappaB in Colonic Mucosa from Patients with Collagenous and Ulcerative Colitis. Gut.

[132] Xia Y., Liu N., Xie X., Bi G., Ba H., Li L., Zhang J., Deng X., Yao Y., Tang Z. (2019). The Macrophage-Specific V-ATPase Subunit ATP6V0D2 Restricts Inflammasome Activation and Bacterial Infection by Facilitating Autophagosome-Lysosome Fusion. Autophagy.

[133] Hossain M.N., Sakemura R., Fujii M., Ayusawa D. (2006). G-Protein γ Subunit GNG11 Strongly Regulates Cellular Senescence. Biochem. Biophys. Res. Commun..

[134] Thierer J.H., Foresti O., Yadav P.K., Wilson M.H., Moll T.O.C., Shen M.C., Busch-Nentwich E.M., Morash M., Mohlke K.L., Rawls J.F. (2024). Pla2g12b Drives Expansion of Triglyceride-Rich Lipoproteins. Nat. Commun..

[135] Schleicher E., Friess U. (2007). Oxidative Stress, AGE, and Atherosclerosis. Kidney Int..

[136] Cannizzaro L., Rossoni G., Savi F., Altomare A., Marinello C., Saethang T., Carini M., Payne D.M., Pisitkun T., Aldini G. (2017). Regulatory Landscape of AGE-RAGE-Oxidative Stress Axis and Its Modulation by PPARγ Activation in High Fructose Diet-Induced Metabolic Syndrome. Nutr. Metab..

[137] Cabrera-García A.I., Suchodolski J.S., Steiner J.M., Heilmann R.M. (2020). Association between Serum Soluble Receptor for Advanced Glycation End-Products (RAGE) Deficiency and Severity of Clinicopathologic Evidence of Canine Chronic Inflammatory Enteropathy. J. Vet. Diagn. Investig..

[138] Cabrera-García A.I., Protschka M., Alber G., Kather S., Dengler F., Müller U., Steiner J.M., Heilmann R.M. (2021). Dysregulation of Gastrointestinal RAGE (Receptor for Advanced Glycation End Products) Expression in Dogs with Chronic Inflammatory Enteropathy. Vet. Immunol. Immunopathol..

[139] Heilmann R.M., Otoni C.C., Jergens A.E., Grützner N., Suchodolski J.S., Steiner J.M. (2014). Systemic Levels of the Anti-Inflammatory Decoy Receptor Soluble RAGE (Receptor for Advanced Glycation End Products) Are Decreased in Dogs with Inflammatory Bowel Disease. Vet. Immunol. Immunopathol..

[140] Fu X.H., Chen C.Z., Wang Y., Peng Y.X., Wang W.H., Yuan B., Gao Y., Jiang H., Zhang J.B. (2019). COL1A1 Affects Apoptosis by Regulating Oxidative Stress and Autophagy in Bovine Cumulus Cells. Theriogenology.

[141] Mao M., Labelle-Dumais C., Keene D.R., Gould D.B. (2022). Elevated TGFβ Signaling Contributes to Ocular Anterior Segment Dysgenesis in Col4a1 Mutant Mice. Matrix Biol..

[142] Faustman D., Davis M. (2010). TNF Receptor 2 Pathway: Drug Target for Autoimmune Diseases. Nat. Rev. Drug Discov..

[143] Blaser H., Dostert C., Mak T.W., Brenner D. (2016). TNF and ROS Crosstalk in Inflammation. Trends Cell Biol..

[144] Defer N., Azroyan A., Pecker F., Pavoine C. (2007). TNFR1 and TNFR2 Signaling Interplay in Cardiac Myocytes. J. Biol. Chem..

[145] Fischer R., Maier O. (2015). Interrelation of Oxidative Stress and Inflammation in Neurodegenerative Disease: Role of TNF. Oxid. Med. Cell Longev..

[146] Fischer R., Maier O., Siegemund M., Wajant H., Scheurich P., Pfizenmaier K. (2011). A TNF Receptor 2 Selective Agonist Rescues Human Neurons from Oxidative Stress-Induced Cell Death. PLoS ONE.

[147] Blume-Jensen P., Janknecht R., Hunter T. (1998). The Kit Receptor Promotes Cell Survival via Activation of PI 3-Kinase and Subsequent Akt-Mediated Phosphorylation of Bad on Ser136. Curr. Biol..

[148] Cardone M.H., Roy N., Stennicke H.R., Salvesen G.S., Franke T.F., Stanbridge E., Frisch S., Reed J.C. (1998). Regulation of Cell Death Protease Caspase-9 by Phosphorylation. Science.

[149] Veroni C., Gabriele L., Canini I., Castiello L., Coccia E., Remoli M.E., Columba-Cabezas S., Aricò E., Aloisi F., Agresti C. (2010). Activation of TNF Receptor 2 in Microglia Promotes Induction of Anti-Inflammatory Pathways. Mol. Cell. Neurosci..

[150] Maier O., Fischer R., Agresti C., Pfizenmaier K. (2013). TNF Receptor 2 Protects Oligodendrocyte Progenitor Cells against Oxidative Stress. Biochem. Biophys. Res. Commun..

[151] Chipuk J.E., Moldoveanu T., Llambi F., Parsons M.J., Green D.R. (2010). The BCL-2 Family Reunion. Mol. Cell.

[152] Baud O., Haynes R.F., Wang H., Folkerth R.D., Li J., Volpe J.J., Rosenberg P.A. (2004). Developmental Up-Regulation of MnSOD in Rat Oligodendrocytes Confers Protection against Oxidative Injury. Eur. J. Neurosci..

[153] Wong G.H.W., Goeddel D.V. (1988). Induction of Manganous Superoxide Dismutase by Tumor Necrosis Factor: Possible Protective Mechanism. Science.

[154] Moianu A., Andone S., Stoian A., Bălașa R., Huțanu A., Sărmășan E. (2024). A Potential Role of Interleukin-5 in the Pathogenesis and Progression of Amyotrophic Lateral Sclerosis: A New Molecular Perspective. Int. J. Mol. Sci..

[155] Hoppenot D., Malakauskas K., Lavinskiene S., Sakalauskas R. (2015). P-STAT6, PU.1, and NF-ΚB Are Involved in Allergen-Induced Late-Phase Airway Inflammation in Asthma Patients. BMC Pulm. Med..

[156] Zhou Y., Li C., Li D., Zheng Y., Wang J. (2017). IL-5 Blocks Apoptosis and Tau Hyperphosphorylation Induced by Aβ25-35 Peptide in PC12 Cells. J. Physiol. Biochem..

[157] Marasco M.R., Conteh A.M., Reissaus C.A., Cupit J.E., Appleman E.M., Mirmira R.G., Linnemann A.K. (2018). Interleukin-6 Reduces B-Cell Oxidative Stress by Linking Autophagy with the Antioxidant Response. Diabetes.

[158] El-Assal O., Hong F., Kim W.H., Radaeva S., Gao B. (2004). IL-6-Deficient Mice Are Susceptible to Ethanol-Induced Hepatic Steatosis: IL-6 Protects against Ethanol-Induced Oxidative Stress and Mitochondrial Permeability Transition in the Liver. Cell. Mol. Immunol..

[159] Matsuoka Y., Nakayama H., Yoshida R., Hirosue A., Nagata M., Tanaka T., Kawahara K., Sakata J., Arita H., Nakashima H. (2016). IL-6 Controls Resistance to Radiation by Suppressing Oxidative Stress via the Nrf2-Antioxidant Pathway in Oral Squamous Cell Carcinoma. Br. J. Cancer.

[160] Wruck C.J., Streetz K., Pavic G., Götz M.E., Tohidnezhad M., Brandenburg L.O., Varoga D., Eickelberg O., Herdegen T., Trautwein C. (2011). Nrf2 Induces Interleukin-6 (IL-6) Expression via an Antioxidant Response Element within the IL-6 Promoter. J. Biol. Chem..

[161] Daffu G., del Pozo C.H., O’Shea K.M., Ananthakrishnan R., Ramasamy R., Schmidt A.M. (2013). Radical Roles for RAGE in the Pathogenesis of Oxidative Stress in Cardiovascular Diseases and Beyond. Int. J. Mol. Sci..

[162] Pathomthongtaweechai N., Chutipongtanate S. (2020). AGE/RAGE Signaling-Mediated Endoplasmic Reticulum Stress and Future Prospects in Non-Coding RNA Therapeutics for Diabetic Nephropathy. Biomed. Pharmacother..

[163] El-Deeb M.M.K., El-Sheredy H.G., Mohammed A.F. (2019). The Possible Role of Interleukin (IL)-18 and Nitrous Oxide and Their Relation to Oxidative Stress in the Development and Progression of Breast Cancer. Asian Pac. J. Cancer Prev..

[164] Ferrer-Torres D., Nancarrow D.J., Steinberg H., Wang Z., Kuick R., Weh K.M., Mills R.E., Ray D., Ray P., Lin J. (2018). Constitutively Higher Level of GSTT2 in Esophageal Tissues From African Americans Protects Cells Against DNA Damage. Gastroenterology.

[165] Preynat-Seauve O., Coudurier S., Favier A., Marche P.N., Villiers C. (2003). Oxidative Stress Impairs Intracellular Events Involved in Antigen Processing and Presentation to T Cells. Cell Stress. Chaperones.

[166] De Rasmo D., Signorile A., Roca E., Papa S. (2009). CAMP Response Element-Binding Protein (CREB) Is Imported into Mitochondria and Promotes Protein Synthesis. FEBS J..

[167] Luciano-Mateo F., Cabré N., Fernández-Arroyo S., Baiges-Gaya G., Hernández-Aguilera A., Rodríguez-Tomàs E., Muñoz-Pinedo C., Menéndez J.A., Camps J., Joven J. (2020). Chemokine C–C Motif Ligand 2 Overexpression Drives Tissue-Specific Metabolic Responses in the Liver and Muscle of Mice. Sci. Rep..

[168] Cescon M., Gattazzo F., Chen P., Bonaldo P. (2015). Collagen VI at a Glance. J. Cell Sci..

[169] Guan Z.-H., Yang D., Wang Y., Ma J.-B., Wang G.-N. (2024). Ectodysplasin-A2 Receptor (EDA2R) Knockdown Alleviates Myocardial Ischemia/Reperfusion Injury through Inhibiting the Activation of the NF-ΚB Signaling Pathway. Exp. Anim..

[170] Hirooka Y., Nozaki Y., Niki K., Inoue A., Sugiyama M., Kinoshita K., Funauchi M., Matsumura I. (2020). Foxp3-Positive Regulatory T Cells Contribute to Antifibrotic Effects in Renal Fibrosis via an Interleukin-18 Receptor Signaling Pathway. Front. Med..

[171] Shang B., Liu Y., Jiang S.J., Liu Y. (2015). Prognostic Value of Tumor-Infiltrating FoxP3+ Regulatory T Cells in Cancers: A Systematic Review and Meta-Analysis. Sci. Rep..

[172] Lin C., Zhao X., Sun D., Zhang L., Fang W., Zhu T., Wang Q., Liu B., Wei S., Chen G. (2016). Transcriptional Activation of Follistatin by Nrf2 Protects Pulmonary Epithelial Cells against Silica Nanoparticle-Induced Oxidative Stress. Sci. Rep..

[173] Kaur G., Wang X., Li X., Ong H., He X., Cai C. (2023). Overexpression of GREM1 Improves the Survival Capacity of Aged Cardiac Mesenchymal Progenitor Cells via Upregulation of the ERK/NRF2-Associated Antioxidant Signal Pathway. Cells.

[174] Hashemi S., Salma J., Wales S., McDermott J.C. (2015). Pro-Survival Function of MEF2 in Cardiomyocytes Is Enhanced by β-Blockers. Cell Death Discov..

[175] She H., Mao Z. (2011). Regulation of Myocyte Enhancer Factor-2 Transcription Factors by Neurotoxins. Neurotoxicology.

[176] Wang F., Wang H., Liu X., Yu H., Huang X., Huang W., Wang G. (2021). Neuregulin-1 Alleviate Oxidative Stress and Mitigate Inflammation by Suppressing NOX4 and NLRP3/Caspase-1 in Myocardial Ischaemia-Reperfusion Injury. J. Cell. Mol. Med..

[177] Fehrenbacher N., Bar-Sagi D., Philips M. (2009). Ras/MAPK Signaling from Endomembranes. Mol. Oncol..

[178] Downward J. (1998). Ras Signalling and Apoptosis. Curr. Opin. Genet. Dev..

[179] Hlavatá L., Nyström T. (2003). Ras Proteins Control Mitochondrial Biogenesis and Function in Saccharomyces Cerevisiae. Folia Microbiol..

[180] Kamp D.W., Shacter E., Weitzman S.A. (2011). Chronic Inflammation and Cancer: The Role of the Mitochondria. Oncology.

[181] Krishnappa V., Boregowda S.V., Phinney D.G. (2014). FGF2 Protects Mouse Mesenchymal Stem Cells from Oxidative Stress by Modulating a Twist2-P53 Signaling Axis. Cytotherapy.

[182] Mei L., Chen Y., Chen P., Chen H., He S., Jin C., Wang Y., Hu Z., Li W., Jin L. (2022). Fibroblast Growth Factor 7 Alleviates Myocardial Infarction by Improving Oxidative Stress via PI3Kα/AKT-Mediated Regulation of Nrf2 and HXK2. Redox Biol..

[183] Zhou D.-P., Deng L.-C., Feng X., Xu H.-J., Tian Y., Yang W.-W., Zeng P.-P., Zou L.-H., Yan X.-H., Zhu X.-Y. (2023). FGF10 Mitigates Doxorubicin-Induced Myocardial Toxicity in Mice via Activation of FGFR2b/PHLDA1/AKT Axis. Acta Pharmacol. Sin..

[184] Gao S., Guo K., Chen Y., Zhao J., Jing R., Wang L., Li X., Hu Z., Xu N., Li X. (2021). Keratinocyte Growth Factor 2 Ameliorates UVB-Induced Skin Damage via Activating the AhR/Nrf2 Signaling Pathway. Front. Pharmacol..

[185] Codocedo J.F., Montecinos-Oliva C., Inestrosa N.C. (2015). Wnt-Related SynGAP1 Is a Neuroprotective Factor of Glutamatergic Synapses against Aβ Oligomers. Front. Cell. Neurosci..

[186] Sato T., Takino J.I., Nagamine K., Nishio K., Hori T. (2019). RASGRP2 Suppresses Apoptosis via Inhibition of ROS Production in Vascular Endothelial Cells. Sci. World J..

[187] Tang S., Chen T., Yu Z., Zhu X., Yang M., Xie B., Li N., Cao X., Wang J. (2014). RasGRP3 Limits Toll-like Receptor-Triggered Inflammatory Response in Macrophages by Activating Rap1 Small GTPase. Nat. Commun..

[188] Bokoch G.M. (2003). Biology of the P21-Activated Kinases. Annu. Rev. Biochem..

[189] Orr A.W., Hahn C., Blackman B.R., Schwartz M.A. (2008). P21-Activated Kinase Signaling Regulates Oxidant-Dependent NF-ΚB Activation by Flow. Circ. Res..

[190] Cunha D.A., Cito M., Carlsson P.O., Vanderwinden J.M., Molkentin J.D., Bugliani M., Marchetti P., Eizirik D.L., Cnop M. (2016). Thrombospondin 1 Protects Pancreatic β-Cells from Lipotoxicity via the PERK–NRF2 Pathway. Cell Death Differ..

[191] Park S.H., Kim J.J., Chung J.S., Lee S.R., Lee G.Y., Kim H.J., Yoo Y. (2011). Do RASSF1A Suppresses the Activated K-Ras-Induced Oxidative DNA Damage. Biochem. Biophys. Res. Commun..

[192] Gordon M., El-Kalla M., Zhao Y., Fiteih Y., Law J., Volodko N., Mohamed A., El-Kadi A.O.S., Liu L., Odenbach J. (2013). The Tumor Suppressor Gene, RASSF1A, Is Essential for Protection against Inflammation -Induced Injury. PLoS ONE.

[193] Chung J., Huda M.N., Shin Y., Han S., Akter S., Kang I., Ha J., Choe W., Choi T.G., Kim S.S. (2021). Correlation between Oxidative Stress and Transforming Growth Factor-Beta in Cancers. Int. J. Mol. Sci..

[194] Son Y., Kim S., Chung H.T., Pae H.O. (2013). Reactive Oxygen Species in the Activation of MAP Kinases. Methods Enzymol..

[195] Domínguez-Pérez M., Nuño-Lámbarri N., Clavijo-Cornejo D., Luna-López A., Souza V., Bucio L., Miranda R.U., Muñoz L., Gomez-Quiroz L.E., Uribe-Carvajal S. (2016). Hepatocyte Growth Factor Reduces Free Cholesterol-Mediated Lipotoxicity in Primary Hepatocytes by Countering Oxidative Stress. Oxid. Med. Cell. Longev..

[196] Clavijo-Cornejo D., Enriquez-Cortina C., López-Reyes A., Domínguez-Pérez M., Nuño N., Domínguez-Meraz M., Bucio L., Souza V., Factor V.M., Thorgeirsson S.S. (2013). Biphasic Regulation of the NADPH Oxidase by HGF/c-Met Signaling Pathway in Primary Mouse Hepatocytes. Biochimie.

[197] López-Ramirez J., Lazzarini-Lechuga R., Gerardo-Ramírez M., Escobedo-Calvario A., Chávez-Rodríguez L., Salas-Silva S., Nuño-Lámbarri N., Massó F., Souza-Arroyo V., Miranda-Labra R.U. (2022). The Hepatocyte Growth Factor Induces an Anti-Inflammatory and Repairing Response in the Cholestasis-Induced Colon Damage. Open Explor..

[198] Asao H., Okuyama C., Kumaki S., Ishii N., Tsuchiya S., Foster D., Sugamura K. (2001). Cutting Edge: The Common Gamma-Chain Is an Indispensable Subunit of the IL-21 Receptor Complex. J. Immunol..

[199] Loschinski R., Böttcher M., Stoll A., Bruns H., Mackensen A., Mougiakakos D. (2018). IL-21 Modulates Memory and Exhaustion Phenotype of T-Cells in a Fatty Acid Oxidation-Dependent Manner. Oncotarget.

[200] Quintero-Villegas A., Valdés-Ferrer S.I. (2019). Role of 5-HT7 Receptors in the Immune System in Health and Disease. Mol. Med..

[201] Yuksel T.N., Yayla M., Halici Z., Cadirci E., Polat B., Kose D. (2019). Protective Effect of 5-HT7 Receptor Activation against Glutamate-Induced Neurotoxicity in Human Neuroblastoma SH-SY5Y Cells via Antioxidative and Antiapoptotic Pathways. Neurotoxicol. Teratol..

[202] Ong Q., Guo S., Duan L., Zhang K., Collier E.A., Cui B. (2016). The Timing of Raf/ERK and AKT Activation in Protecting PC12 Cells against Oxidative Stress. PLoS ONE.

[203] Zheng L., Ishii Y., Tokunaga A., Hamashima T., Shen J., Zhao Q.L., Ishizawa S., Fujimori T., Nabeshima Y.I., Mori H. (2010). Neuroprotective Effects of PDGF against Oxidative Stress and the Signaling Pathway Involved. J. Neurosci. Res..

[204] Hartman E.S., Brindley E.C., Papoin J., Ciciotte S.L., Zhao Y., Peters L.L., Blanc L. (2018). Increased Reactive Oxygen Species and Cell Cycle Defects Contribute to Anemia in the RASA3 Mutant Mouse Model Scat. Front. Physiol..

[205] Weng M.S., Chang J.H., Hung W.Y., Yang Y.C., Chien M.H. (2018). The Interplay of Reactive Oxygen Species and the Epidermal Growth Factor Receptor in Tumor Progression and Drug Resistance. J. Exp. Clin. Cancer Res..

[206] Mak I.T., Kramer J.H., Chmielinska J.J., Spurney C.F., Weglicki W.B. (2015). EGFR-TKI, Erlotinib, Causes Hypomagnesemia, Oxidative Stress and Cardiac Dysfunction: Attenuation by NK-1 Receptor Blockade. J. Cardiovasc. Pharmacol..

[207] Coskun M., Salem M., Pedersen J., Nielsen O.H. (2013). Involvement of JAK/STAT Signaling in the Pathogenesis of Inflammatory Bowel Disease. Pharmacol. Res..

[208] Banerjee S., Biehl A., Gadina M., Hasni S., Schwartz D.M. (2017). JAK–STAT Signaling as a Target for Inflammatory and Autoimmune Diseases: Current and Future Prospects. Drugs.

[209] Manz A., Allenspach K., Kummer S., Richter B., Walter I., Macho-Maschler S., Tichy A., Burgener I.A., Luckschander-Zeller N. (2021). Upregulation of Signal Transducer and Activator of Transcription 3 in Dogs with Chronic Inflammatory Enteropathies. J. Vet. Intern. Med..

[210] Richards C.D. (2013). The Enigmatic Cytokine Oncostatin M and Roles in Disease. ISRN Inflamm..

[211] Werner S.L., Sharma R., Woodruff K., Horn D., Harris S.E., Gorin Y., Lee D.Y., Hua R., Gu S., Fajardo R.J. (2020). CSF-1 in Osteocytes Inhibits Nox4-Mediated Oxidative Stress and Promotes Normal Bone Homeostasis. JBMR Plus.

[212] Kojima H., Otani A., Oishi A., Makiyama Y., Nakagawa S., Yoshimura N. (2011). Granulocyte Colony-Stimulating Factor Attenuates Oxidative Stress–Induced Apoptosis in Vascular Endothelial Cells and Exhibits Functional and Morphologic Protective Effect in Oxygen-Induced Retinopathy. Blood.

[213] Singh B., Kosuru R., Lakshmikanthan S., Sorci-Thomas M.G., Zhang D.X., Sparapani R., Vasquez-Vivar J., Chrzanowska M. (2021). Endothelial Rap1 (Ras-Association Proximate 1) Restricts Inflammatory Signaling to Protect From the Progression of Atherosclerosis. Arterioscler. Thromb. Vasc. Biol..

[214] Yang Z., Kirton H.M., Al-Owais M., Thireau J., Richard S., Peers C., Steele D.S. (2017). Epac2-Rap1 Signaling Regulates Reactive Oxygen Species Production and Susceptibility to Cardiac Arrhythmias. Antioxid. Redox Signal..

[215] Wang H., Jiang Y., Shi D., Quilliam L.A., Chrzanowska-Wodnicka M., Wittchen E.S., Li D.Y., Hartnett M.E. (2014). Activation of Rap1 Inhibits NADPH Oxidase-Dependent ROS Generation in Retinal Pigment Epithelium and Reduces Choroidal Neovascularization. FASEB J..

[216] Gertzberg N., Neumann P., Rizzo V., Johnson A. (2004). NAD(P)H Oxidase Mediates the Endothelial Barrier Dysfunction Induced by TNF-Alpha. Am. J. Physiol. Lung Cell. Mol. Physiol..

[217] Glaviano A., Foo A.S.C., Lam H.Y., Yap K.C.H., Jacot W., Jones R.H., Eng H., Nair M.G., Makvandi P., Geoerger B. (2023). PI3K/AKT/MTOR Signaling Transduction Pathway and Targeted Therapies in Cancer. Mol. Cancer.

[218] Chiang J.C., Chen W.M., Newman C., Chen B.P.C., Lee H. (2022). Lysophosphatidic Acid Receptor 3 Promotes Mitochondrial Homeostasis against Oxidative Stress: Potential Therapeutic Approaches for Hutchinson-Gilford Progeria Syndrome. Antioxidants.

[219] Djavaheri-Mergny M., Javelaud D., Wietzerbin J., Besançon F. (2004). NF-KappaB Activation Prevents Apoptotic Oxidative Stress via an Increase of Both Thioredoxin and MnSOD Levels in TNFalpha-Treated Ewing Sarcoma Cells. FEBS Lett..

[220] Thimmulappa R.K., Lee H., Rangasamy T., Reddy S.P., Yamamoto M., Kensler T.W., Biswal S. (2016). Nrf2 Is a Critical Regulator of the Innate Immune Response and Survival during Experimental Sepsis. J. Clin. Investig..

[221] Greco L., Gobbetti M., Auricchio R., Di Mase R., Landolfo F., Paparo F., Di Cagno R., De Angelis M., Rizzello C.G., Cassone A. (2011). Safety for Patients with Celiac Disease of Baked Goods Made of Wheat Flour Hydrolyzed During Food Processing. Clin. Gastroenterol. Hepatol..

[222] Von Berg A., Koletzko S., Grübl A., Filipiak-Pittroff B., Wichmann H.E., Bauer C.P., Reinhardt D., Berdel D. (2003). The Effect of Hydrolyzed Cow’s Milk Formula for Allergy Prevention in the First Year of Life: The German Infant Nutritional Intervention Study, a Randomized Double-Blind Trial. J. Allergy Clin. Immunol..

[223] Mandigers P.J.J., Biourge V., Van Den Ingh T.S.G.A.M., Ankringa N., German A.J. (2010). A Randomized, Open-Label, Positively-Controlled Field Trial of a Hydrolyzed Protein Diet in Dogs with Chronic Small Bowel Enteropathy. J. Vet. Intern. Med..

[224] Cave N.J. (2006). Hydrolyzed Protein Diets for Dogs and Cats. Vet. Clin. N. Am.-Small Anim. Pract..

[225] Olivry T., Bizikova P. (2010). A Systematic Review of the Evidence of Reduced Allergenicity and Clinical Benefit of Food Hydrolysates in Dogs with Cutaneous Adverse Food Reactions. Vet. Dermatol..

[226] Ambrosini Y.M., Neuber S., Borcherding D., Seo Y.J., Segarra S., Glanemann B., Garden O.A., Müller U., Adam M.G., Dang V. (2020). Treatment with Hydrolyzed Diet Supplemented with Prebiotics and Glycosaminoglycans Alters Lipid Metabolism in Canine Inflammatory Bowel Disease. Front. Vet. Sci..

