# Supplementary figures and images for: Restorative Effects of Synbiotics on Colonic Ultrastructure and Oxidative Stress in Dogs with Chronic Enteropathy
Source: Antioxidants (Basel). 2025 Jun 13;14(6):727. doi: 10.3390/antiox14060727 (PMC12189513; doi:10.3390/antiox14060727)

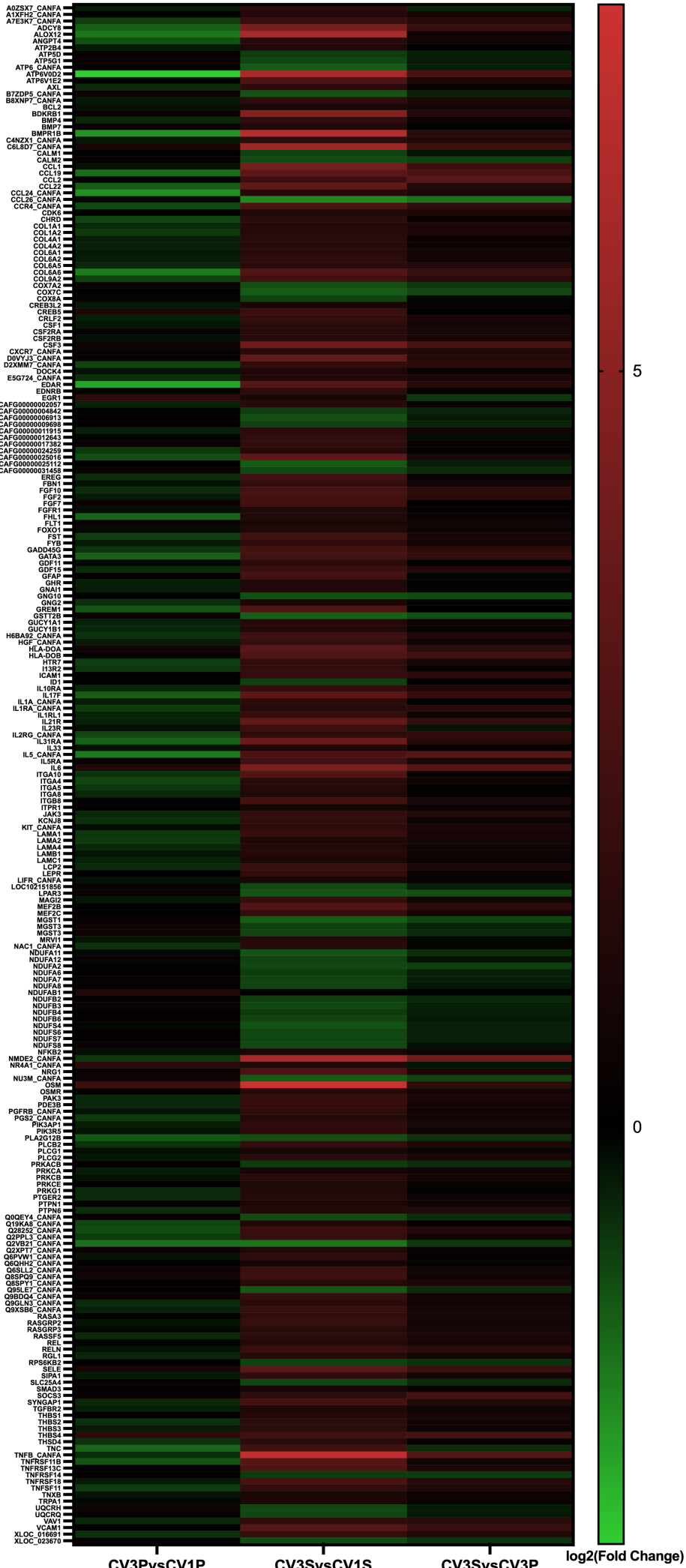

CV3PvsCV1P CV3SvsCV1S CV3SvsCV3P log2(Fold Change)

Supplement: Supplementary file 1 [file antioxidants-14-00727-s001.zip › antioxidants-3618356-supplementary.pdf]
